# Evaluation of Non-Uniform Sampling 2D ^1^H–^13^C HSQC Spectra for Semi-Quantitative Metabolomics

**DOI:** 10.3390/metabo10050203

**Published:** 2020-05-16

**Authors:** Bo Zhang, Robert Powers, Elizabeth M. O’Day

**Affiliations:** 1 Olaris, Inc., Waltham, MA 02451, USA; bzhang@olarisbor.com; 2 Department of Chemistry, University of Nebraska-Lincoln, Lincoln, NE 68588-0304, USA; rpowers3@unl.edu; 3 Nebraska Center for Integrated Biomolecular Communication, University of Nebraska-Lincoln, Lincoln, NE 68588-0304, USA

**Keywords:** metabolomics, NUS NMR, reproducibility

## Abstract

Metabolomics is the comprehensive study of metabolism, the biochemical processes that sustain life. By comparing metabolites between healthy and disease states, new insights into disease mechanisms can be uncovered. NMR is a powerful analytical method to detect and quantify metabolites. Standard one-dimensional (1D) ^1^H-NMR metabolite profiling is informative but challenged by significant chemical shift overlap. Multi-dimensional NMR can increase resolution, but the required long acquisition times lead to limited throughput. Non-uniform sampling (NUS) is a well-accepted mode of acquiring multi-dimensional NMR data, enabling either reduced acquisition times or increased sensitivity in equivalent time. Despite these advantages, the technique is not widely applied to metabolomics. In this study, we evaluated the utility of NUS ^1^H–^13^C heteronuclear single quantum coherence (HSQC) for semi-quantitative metabolomics. We demonstrated that NUS improved sensitivity compared to uniform sampling (US). We verified that the NUS measurement maintains linearity, making it possible to detect metabolite changes across samples and studies. Furthermore, we calculated the lower limit of detection and quantification (LOD/LOQ) of common metabolites. Finally, we demonstrate that the measurements are repeatable on the same system and across different systems. In conclusion, our results detail the analytical capability of NUS and, in doing so, empower the future use of NUS ^1^H–^13^C HSQC in metabolomic studies.

## 1. Introduction

Metabolomics is the global measurement of metabolism, the biochemical processes that allow organisms to grow, reproduce, maintain their structures, and respond to genetic and environmental factors [1,2,3]. In its simplest form, metabolism is the set of pathways used to both build (anabolism) and breakdown (catabolism) the macromolecules that comprise all living matter [4]. Metabolism plays important roles in cellular communication, signaling, and regulation [5]. Characterizing and investigating metabolism is, therefore, a means to understand the molecular pathways that support life [6]. Alterations in metabolism were shown to underly many diseases such as diabetes [7], neurodegeneration [8], cancer [9], hepatotoxicity [10], obesity [11], cardiovascular disease [12], inflammation [13], and even aging [14]. Thus, through metabolomics, differences between healthy and diseased states can be uncovered, leading to new insights to diagnose and treat disease.

Metabolites include endogenous metabolites, microbiome byproducts, and xenobiotics. These small molecules span a large chemical space and broad dynamic range [15]. In human plasma, estimates suggest that metabolite concentrations span 12 orders of magnitude (femtomolar to millimolar) [16]. The goal of metabolomics is to identify and to quantify the complete set of metabolites in a highly reproducible manner [17]. The two main analytical platforms in metabolomics are liquid chromatography and/or gas chromatography coupled to mass spectrometry (MS) and nuclear magnetic resonance spectroscopy (NMR) [18]. MS is a popular detector due to its high resolution and sensitivity, which leads to a broad coverage of the metabolome [19]. However, metabolite identification based on mass remains challenging [20]. Lack of reproducibility is also a major obstacle for MS, wherein significant variations in metabolite measurements can occur even within the same experiment. Accordingly, comparisons of metabolite changes and longitudinal studies are difficult to accomplish with MS [21]. On the other hand, NMR measurements are highly reproducible. NMR also provides comprehensive, atomic-resolution structural information, which greatly improves the accuracy of metabolite assignments. Notably, NMR accomplishes this without the need for chromatography, which avoids sample bias [22,23]. However, NMR suffers from low sensitivity and low resolution [24]. 

Multi-dimensional NMR spectroscopy increases resolution and reduces signal overlap by dispersing resonances into two or more chemical shift dimensions benefiting both identification and quantification. For example, two-dimensional (2D) ^1^H–^13^C heteronuclear single quantum coherence (HSQC) spectroscopy spreads NMR resonances into ^1^H and ^13^C chemical shift dimensions. The HSQC experiment enables the detection of all directly bonded H–C pairs in a metabolite [25]. For metabolite identification, experimental HSQC data can be searched against reference libraries, such as the Human Metabolome Database (HMDB) [26], Biological Magnetic Resonance Bank (BMRB) [27] and Complex Mixture Analysis by NMR (COLMAR) [28], which contain ^1^H and ^13^C chemical shift information for hundreds to thousands of known metabolites. Although the HSQC experiment can enhance resolution, the sensitivity issue is worsened. This is due, in part, to the low 1% natural abundance of ^13^C. As a result, an HSQC spectrum requires extremely long acquisition times to obtain a sufficient signal-to-noise ration. Employing traditional 2D ^1^H–^13^C HSQC experiments for metabolite profiling on the large number of samples typically required for metabolomics is de facto impossible [29]. 

Non-uniform sampling (NUS) is a well-established mode of acquiring multi-dimensional NMR data [30,31]. Instead of uniformly sampling (US) data during the entire acquisition period, NUS acquires a subset of measurements between the first and last time points. The full dataset is generated using reconstruction algorithms [31]. Since fewer measurements are collected, experimental time is greatly reduced. Previous studies also demonstrated that NUS contributes to the reproducibility of the spectra [32,33,34]. When employed properly, NUS can generate highly reproducible and quantitative spectra in a reduced amount of time. It was previously demonstrated that NUS applied to homonuclear NMR metabolite profiling (^1^H–^1^H-TOCSY and ^1^H, ^1^H-COSY45) maintains sufficient quantitative accuracy and precision [35]. Accordingly, NUS ^1^H–^13^C HSQC could be a powerful technique for metabolomics, especially for large datasets. Indeed, pivotal studies demonstrated the feasibility of applying NUS-HSQC to metabolomics. NUS was used with a J-compensated quantitative HSQC pulse sequence and an 800-MHz NMR, which led to a 22-fold reduction in NMR data collection time without compromising the quantitative information of urine metabolites [36]. Encouragingly, NUS spectra also demonstrated a linear response over a range of metabolite concentrations [35,36]. Furthermore, a separate study showed that US and NUS HSQC data generated similar statistical models using synthetic samples designed to mimic the serum of patients affected by colorectal cancer patients [37]. It is important to note that the metabolite concentrations in the synthetic samples were enhanced by a factor of 20 compared to authentic serum samples. Accordingly, both studies only focused on the identification and quantification of a few select metabolites at millimolar concentrations. Thus, the ability of NUS HSQC to accurately characterize metabolomics profiles for a chemically diverse set of metabolites over a wide concentration range was not rigorously investigated. 

In this study, we evaluated the utility of NUS ^1^H–^13^C HSQC for semi-quantitative metabolomics. We provide further confirmation that NUS improved sensitivity compared to US sampling. We verified that NUS measurements maintain linearity, making it possible to detect metabolite changes across samples and studies. Furthermore, we calculated the lower limit of detection (LOD) and the lower limit of quantification (LOQ) of common metabolites (low µM) using NUS ^1^H–^13^C HSQC. Furthermore, we systematically tested the reproducibility of NUS measurements on the same magnet and across magnets to establish a set of guidelines for future NUS ^1^H–^13^C HSQC metabolomic studies. 

## 2. Results

### 2.1. NUS Provides Enhanced Sensitivity

**Figure 1:**
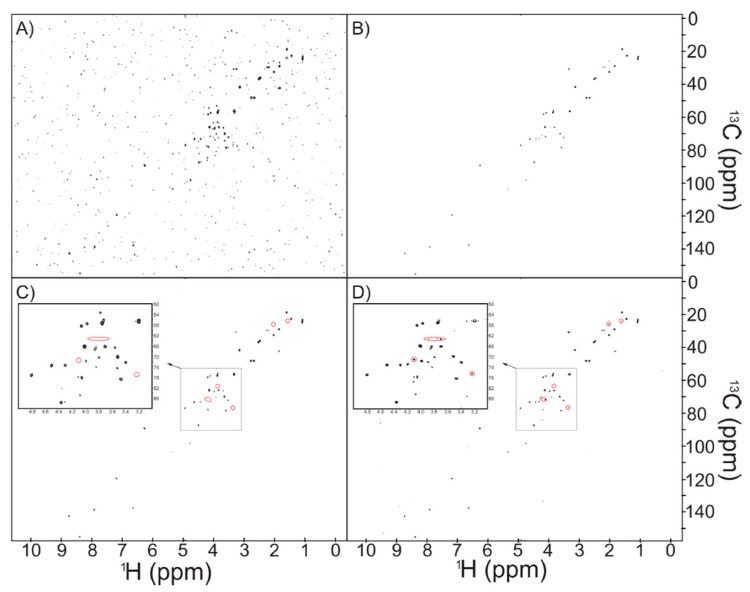
Non-uniform sampling (NUS) increases sensitivity and resolution compared to uniform sampling (US). US (**A**) and NUS (**B**) spectra for Reference 1 when the maximum peak intensity of each spectra is normalized to 10. US (**C**) and NUS (**D**) spectra for Reference 1 at the same contour level. Red circles indicate resonances only detected in the NUS spectrum.

The acquisition time required for traditional multidimensional NMR represents a significant bottleneck for metabolomics. NUS offers a potential solution by collecting fewer increments in the indirect dimension and then reconstructing the full spectrum. In this regard, it is possible to either decrease the overall experiment time or increase the *apparent* signal-to-noise ratio by increasing the number of scans. A reference mixture (“Reference 1”) composed of 15 common human metabolites at 500 µM was used to compare the sensitivity of US vs. NUS (Figure 1 and Figure S3, Supplementary Materials). For the same one-hour acquisition time, the NUS spectrum provides well-resolved resonances for all 15 metabolites. Conversely, the US spectrum contained clearly lower peak intensities. Directly comparing the two spectra indicates an average increase in signal-to-noise ratio (S/N) of 5.86 ± 0.83 in the NUS spectrum. This is better than the expected increase associated with 4× the number of scans. It is important to note that reconstruction methods are non-linear, which may suppress the appearance of noise. Thus, the increased S/N is not a pure reflection of enhanced sensitivity but rather demonstrates that, under these conditions, NUS is more fit for purpose. Notably, both the peak intensity and the noise levels in the NUS spectra were higher than the US spectra. By normalizing the US and NUS peaks to the maximum peak intensity, the enhanced sensitivity of NUS is demonstrated in Figure 1A,B. The US spectrum is overrun with noise, while all of the metabolite NMR resonances are clearly identifiable in the NUS spectrum. Additionally, many peaks are below the noise level (Figure 1C,D) in the US spectrum. Examples of resonances that were only detected in the NUS spectrum are indicated by the red circles. This comparison demonstrates that a NUS HSQC spectrum will yield informative and well-resolved metabolite spectral information in a relatively short amount of time (1 h). Of note, more artefacts were present in NUS compared to US (Figure S3, Supplementary Materials). The NUS reconstruction poses a significant challenge in distinguishing between real metabolite resonances and artefacts. However, metabolomics studies often involve a large number of samples, which should have non-equivalent artefacts. By carefully building statistical models, it is possible to reduce the influence of artefacts. Furthermore, a representative US spectrum could be recorded to validate peak positions. 

### 2.2. NUS Data Are Highly Linear

**Figure 2:**
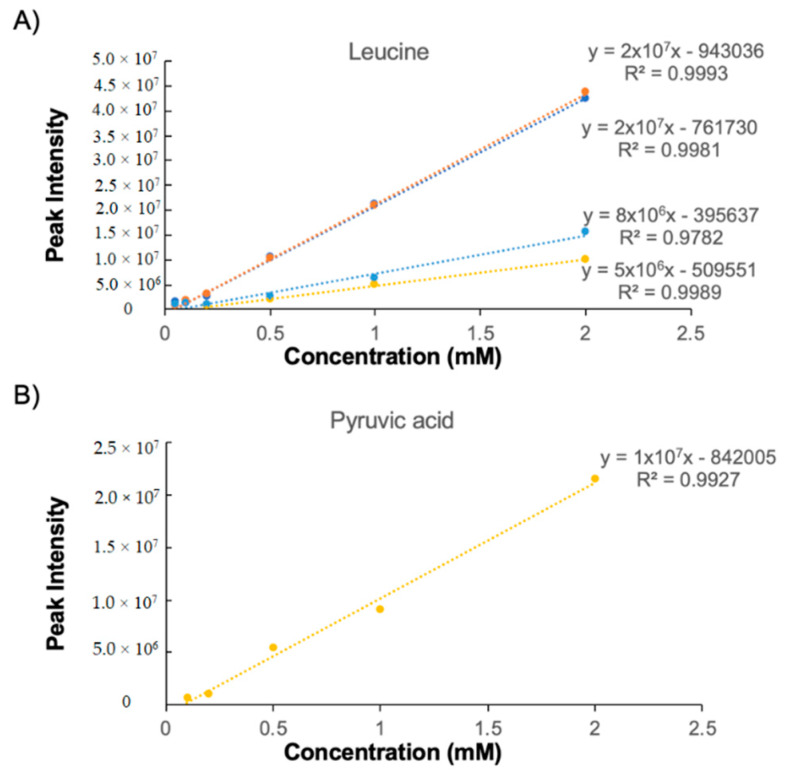
NUS HSQC peak intensity is linear with concentration. Resonance intensity for leucine which has four resonances (**A**) and pyruvic acid which has a single resonance (**B**) increases as a function of metabolite concentration. The best-fit line for each resonance is described.

NMR is a highly quantitative measurement. In one-dimensional (1D) ^1^H-NMR, peak area is proportional to the number of protons in a molecule, enabling complete quantification by adding one or more internal standards of known concentration to a sample. For 2D ^1^H–^13^C HSQC, the ratio of peak area is not a simple 1:1 conversion to concentration due to different coupling constants, relaxation properties, and the number of attached hydrogens for each C–H pair. Nevertheless, for a given peak, intensity correlates with concentration. Thus, it is possible to monitor metabolite concentration changes across multiple samples or studies by using peak intensity for semi- quantification. Rai and colleagues [36] previously demonstrated that, in the presence of the relaxation enhancement reagent Cu(EDTA), NUS HSQC peak intensity was observed to be linear as a function of concentration (~24 to 78 mM) for four amino acids (glycine, alanine, valine, and methionine). We sought to expand upon these preliminary results by testing NUS linearity on an increased number of metabolites that spanned a variety of chemical classes and that covered a larger concentration range (0.05 to 2 mM). Importantly, our analysis of NUS HSQC linearity did not include the addition of any relaxation agents. A series of six NUS HSQC spectra were recorded for a mixture containing 28 metabolites (Reference 2) with concentrations ranging from 50 µM to 2 mM (Table S3, Supplementary Materials). For each resonance, the peak intensity was plotted as a function of concentration and the data were fitted to a linear regression model (Figure S4, Supplementary Materials). Example plots of the four resonance peaks for leucine and the single resonance for pyruvic acid are shown in Figure 2. Overlapped peaks were excluded from the analysis, and the full list of peaks used in the calibrations is provided in Table S5 (Supplementary Materials). More than 98% of metabolite resonances displayed a correlation coefficient of *R^2^* > 0.9, indicating excellent linearity (Table 1). Glucose resonances displayed the lowest *R^2^* values, which is likely due to isomers and the tendency to exchange conformations. Overall, this analysis demonstrates that NUS ^1^H–^13^C HSQC peak intensity data are highly linear as a function of metabolite concentration. 

### 2.3. LOD and LOQ

We next sought to determine the lower limit of detection (LOD) and the lower limit of quantification (LOQ) for our NUS platform, see Equation (1). LOD and LOQ are defined as follows:

LOD = 3 × σ and LOQ = 10 × σ,(1)

where the variance of the noise (σ) was estimated by the median absolute deviation (MAD). MAD was calculated from the COLMAR database, where the positive values of all non-peak data X_i_ were used in the following equations (2) and (3):

*MAD* = *median_i_*(|*X_i_−median_i_(X_j_*)|), (2)

σ = 1.4826 × MAD,(3)

Table 2 and Table 3 list the LOD and LOQ for each of the resonances detected for the 29 metabolites in Reference 2. Metabolites with multiple resonances have an LOD/LOQ for each observed peak and, therefore, metabolites with multiple peaks will have a range of LOD/LOQ. The LOD and LOQ for the majority of metabolites varied from 10 to 30 µM and 50 to 90 µM, respectively. Using these NUS ^1^H–^13^C HSQC experimental conditions, it is possible to detect and quantify metabolites in the low µM range. 

### 2.4. Repeatability of NUS

**Figure 3:**
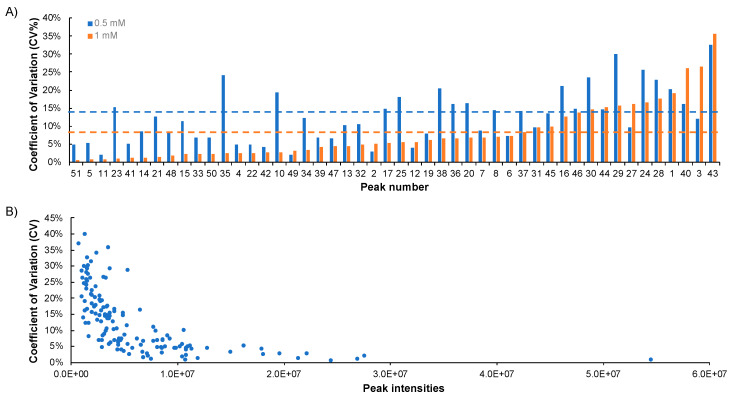
Variance of NUS intensity measurements. (**A**) Coefficient of variation (CV) for all metabolite resonances in Reference 1 at 0.5 mM (blue) and 1 mM (orange). The average CV is provided by the dotted lines. (**B**) Correlation between %CV and peak intensities for all metabolite resonances of Reference 1 at 0.5 mM and 1 mM.

Metabolite profiling requires the reliable and repeatable comparison of results across multiple samples. Prior studies demonstrated the repeatability of 1D ^1^H-NMR and 2D US HSQC experiments [35,38]. To demonstrate a similar repeatability for NUS HSQC experiments, the percent coefficients of variation (%CV) were measured for a dataset of NUS HSQC experiments. Specifically, three to five replicate NUS HSQC spectra were collected for the Reference 1 mixture, which contained 15 metabolites at a concentration of either 500 µM or 1 mM (Figure 3A). The mean %CV was 14% ± 9% and 8% ± 8%, respectively. The %CV range for both samples was 0% to 35%. These results suggest highly reproducible data. Similar results were reported for homonuclear NUS experiments (^1^H–^1^H TOCSY and ^1^H–^1^H COSY), wherein Schlippenbach and colleagues [35] noted a 5 to 22 %CV range for six metabolites spiked into urine samples. 

The decrease in %CV for the 1 mM sample is expected due to the increased concentration and the corresponding increase in peak intensities. As shown in Figure 3B, peak intensity and %CV are inversely related. This suggests a lower limit in peak intensity or signal-to-noise ratio may be used to ensure reproducible data and define an acceptable CV target. For example, the mean %CV of the 50 most intense peaks (peak intensity cut-off of 1.3 × 10^7^) was 4% ± 3%. Dropping the intensity cut-off to 2.0 × 10^6^ exhibited a mean %CV of 21% ± 8%. Based on these observations, we recommend a minimum peak threshold of 2.0 × 10^6^ to maximize data reliability. 

### 2.5. Stability of NUS

**Figure 4:**
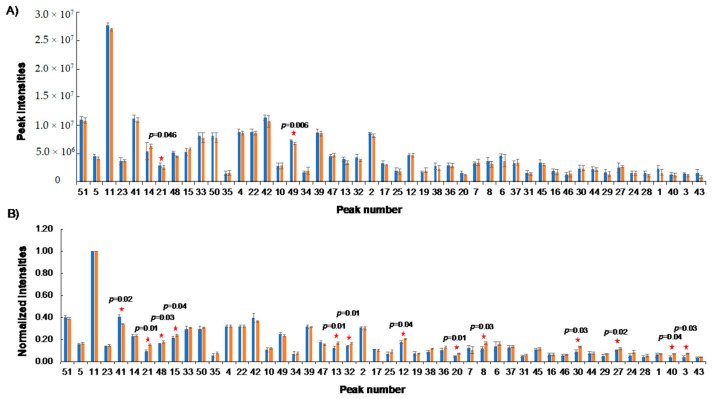
NUS HSQC is highly stable. (**A**) Comparison between absolute peak intensities of two sets of replicate spectra collected 21 days apart. Two peaks with significant difference in intensity (*p* < 0.05) are marked with a star. (**B**) Comparison between normalized peak intensities of two sets of replicate spectra collected on two 600-MHz NMR instruments. Significant differences in intensity (*p* < 0.05) are marked with a star.

To assess the stability of the NUS HSQC peak intensity measurements, a series of NUS HSQC spectra were acquired over a period of 21 days. The Reference 1 mixture at 500 µM was used to measure and compare overall peak intensities (Figure 4A). The intensity difference between the majority of peaks was negligible. Only two lysine peak intensities were significantly different (*p*-value < 0.05) between the time points. However, both peaks displayed relatively small changes in intensity of 7.5% and 15.2%, respectively. Overall, NUS HSQC measurements are highly stable.

### 2.6. NUS Measurements across Systems

We next sought to compare NUS HSQC data collected on different NMR instruments. Using the same 500 µM Reference 1, we recorded NUS HSQC spectra on a 600-MHz NMR with an updated TCI probe cooled by liquid helium and on a 600-MHz NMR TXI probe cooled by liquid nitrogen. Due to the enhanced sensitivity of the TCI probe relative to the TXI probe, each peak in one spectrum was normalized with a maximum of 1 before they were averaged and compared in Figure 4B. The TCI probe yielded a median %CV of 7% ± 6% and a similar range of 0% to 30% (Figure S5, Supplementary Materials). This is slightly improved compared to the %CV observed on the TXI probe of 14% ± 9%. Approximately 13 out of 48 peaks were significantly changed (*p* < 0.05) between the TCI and TXI systems; however, the overall change in intensity was small with an average intensity change of only ~19%. The increased sensitivity of the TXI probe could account for these differences. From this analysis, we can conclude that similar, but not exact, results are obtained on different instruments at the same magnet strength. 

### 2.7. NUS on Plasma Sample

The NUS HSQC experiment was further assessed as a tool for semi-quantitative metabolite profiling by using standard plasma samples. NIST SRM 1950 is a commercially available reference sample designed to represent “normal” human plasma. It was constructed by pooling plasma from 100 individuals (equal distribution of men and women aged 40 to 50) who underwent an overnight fast prior to a blood draw. Using methods described previously, metabolites were extracted from a 1-mL aliquot of NIST SRM 1950 to record a series 2D ^1^H–^13^C HSQC spectra.

**Figure 5:**
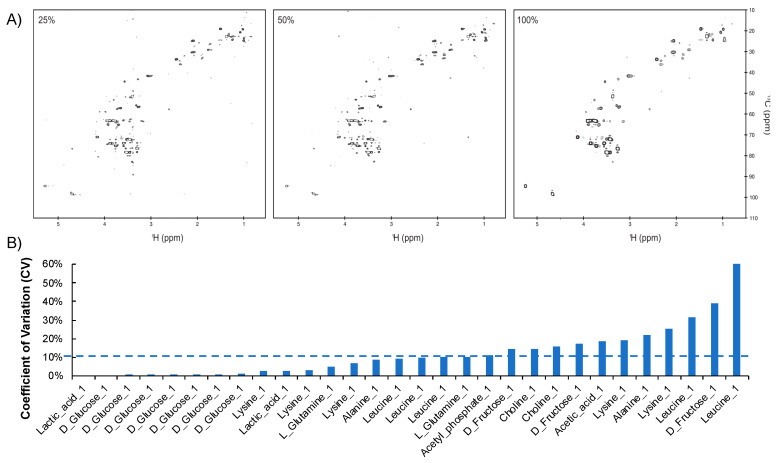
(**A**) NUS HSQC recapitulates US spectra of plasma: Left, 25% NUS (1 h); Middle, 50% NUS (2 h); Right, US (100%) HSQC spectra of an NIST plasma sample. (**B**) Variance of NUS intensity measurements on a plasma sample. %CV of peak intensity in 25% NUS HSQC spectra for select metabolites in the NIST plasma sample. The average CV is provided by the dotted line.

A US HSQC spectrum was acquired with 36 scans and with 512 and 128 complex points in the direct and indirect dimensions, respectively. The total acquisition time for this experiment was 4 h. Additional HSQC spectra were collected with either 50% or 25% NUS (64 or 32 points in the indirect dimension). The acquisition times were reduced to 2 h or 1 h (Figure 5A). The NUS datasets were reconstructed with iterative soft thresholding (IST). A total of 102 resonances were detected above a threshold of 3 × 10^5^ for the US HSQC spectrum. In comparison, 75 and 69 of the 102 peaks were detected for the 50% and 25% NUS HSQC spectra, respectively (Table S6, Supplementary Materials). Applying the recommended 2 × 10^6^ intensity cut-off for robust data (%CV < 20%), the 25% NUS HSQC spectrum was able to recapitulate all of the resonances in the US spectrum, while requiring only one-fourth of the time. In future experiments, acquisition time, sampling density, and intensity cut-off can be tuned to produce robust NUS HSQC spectra that also meet the experimental needs. The %CV for all known metabolites from triplicate NUS HSQC spectra of plasma was determined to be 12% ± 14% (Figure 5B). Collectively, these results support the utility and reproducibility of NUS HSQC data for real-world metabolomic samples. 

## 3. Materials and Methods 

### 3.1. Sample Preparation

The following metabolites were used to generate model mixtures Reference 1 and Reference 2 (Tables S1 and S3, Supplementary Materials): acetylcholine chloride (C_7_H_15_NO_2_∙HCl, >99%), adenosine 5-monophosphate disodium (C_10_H_12_N_5_Na_2_O_7_P, >99%), l-arginine (C_6_H_14_N_4_O_2_, >98%), choline chloride (C_5_H_13_NO∙HCl, >99%), cytidine (C_9_H_13_N_3_O_5_, >99%), d-alpha-hydroxyglutaric acid disodium salt (C_5_H_6_Na_2_O_5_, >98%), alpha-ketoglutaric acid disodium salt dihydrate (C_5_H_4_Na_2_O_5_∙2H_2_O, >98%), beta-nicotinamide adenine dinucleotide hydrate (C_21_H_27_N_7_O_14_P_2_∙*x*H_2_O, >98%), d-(−)-fructose (C_6_H_12_O_6_, >99%), d-(+)-glucosamine hydrochloride (C_6_H_13_NO_5_∙HCl, >99%), guanosine 5-triphosphate sodium salt (C_10_H_16_N_5_O_14_P_3_∙ *x*Na+*y*H_2_O, >95%), lithium potassium acetyl phosphate (C_2_H_3_KLiO_5_P, >97%), dl-malic acid (C_4_H_6_O_5_, >99%), d-ribose 5-phosphate disodium salt dihydrate (C_5_H_9_Na_2_O_8_P∙2H_2_O, >99%), sodium succinate dibasic hexahydrate (C_4_H_4_Na_2_O_4_∙6H_2_O, >99%), sodium acetate (C_2_H_3_NaO_2_, >99%), sodium l-lactate (C_3_H_5_NaO_3_, >99), sodium citrate tribasic dihydrate (C_6_H_5_O_7_Na_3_∙2H_2_O, >99%), sodium fumarate dibasic (C_4_H_2_Na_2_O_4_, >98%), sodium pyruvate (C_3_H_3_NaO_3_, >99%), uridine 5-diphosphate (C_9_H_12_N_2_Na_2_O_12_P_2_∙*x*H_2_O, >96%), l-alanine (C_3_H_7_NO_2_, >98%), l-cysteine (C_3_H_7_NO_2_S, >98%), d-(+)-glucose (C_6_H_12_O_6_, >99.5%), l-glutamic acid monosodium salt monohydrate (C_5_H_8_NNaO_4_∙H_2_O, >99%), l-glutamine (C_5_H_10_N_2_O_3_, >99%), l-histidine (C_6_H_11_N_3_O_3_∙HCl, >98.5%), l-leucine (C_6_H_13_NO_2_, >98.5%), l-lysine, monohydrochloride (C_6_H_14_N_2_O_2_∙HCl, >98.5%), and l-ornithine hydrochloride (C_5_H_12_N_2_O_2_∙HCl, >98%). All the compounds were obtained from Sigma-Aldrich. Deuterium oxide (D_2_O, 99.0%) was purchased from Cambridge Isotope Laboratory, Inc., Andover, MA, USA. 

### 3.2. NMR Sample Preparation

Reference 1 was prepared in 200 µL of 20 mM sodium phosphate buffer at pH 7.4 (uncorrected) in D_2_O. Reference 2 was prepared in 200 µL of 50 mM sodium phosphate buffer at pH 7.4 (uncorrected) in D_2_O. The buffering capacity was increased for Reference 2 because of the higher metabolite concentrations and ionic strength. Samples were transferred to 3-mm NMR tubes. 

Then, 1 mL NIST plasma extract was prepared using methanol/chloroform extraction. The aqueous phase was transferred to a 15-mL Falcon tube and freeze dried. The powder was reconstituted in 200 µL of 20 mM phosphate buffer at pH 7.4 (uncorrected) in D_2_O and then transferred to a 3-mm NMR tube immediately before data collection. 

### 3.3. NMR Experiments and Processing

All NMR spectra were acquired on a Bruker AVANCE II solution-state NMR spectrometer equipped with a liquid-nitrogen-cooled prodigy TXI cryoprobe at 600-MHz proton frequency. A comparative dataset was collected on a Bruker AVANCE III solution-state NMR spectrometer equipped with a liquid-helium-cooled prodigy TCI cryoprobe at 600-MHz proton frequency. NUS schedules were generated using a Poisson gap distribution with a sinusoidal weight of 2 and random seed generator [31]. The same 25% NUS schedule and seed were used for all experiments. All NMR spectra were collected at 298 K. 

The spectral width along the direct and the indirect dimensions were 9578.544 and 24,131.775 Hz, respectively. The number of complex points in the direct dimension was 512 and varied from 32 (25% NUS) to 128 (100% US) for the indirect dimension depending on the NUS sampling density. The number of scans was 36. The transmitter frequency offset was 75 ppm in the ^13^C dimension and 4.7 ppm in the ^1^H dimension. 

NUS data were reconstructed using iterative soft thresholding according to the hmsIST algorithm [31] to generate the same number of direct dimension data points and twice the indirect dimension data points 512 (N_2_) × 256 (N_1_). The reconstructed and US spectral data were then processed with NMRPipe [39]. Both the NUS and US NMR data were zero-filled (two zero-fillings in the direct dimension and three zero-fillings in the indirect dimension), Fourier-transformed, and manually phase-corrected, yielding a final digital resolution of 2048 (N_2_) × 2048 (N_1_) points. Chemical shift queries and metabolite quantifications were performed using the COLMARm NMR webserver (http://spin.ccic.ohio-state.edu/index.php/colmar) [28]. The metabolite lists and assignments were presented for Reference 1 (Tables S1 and S2, Figure S1, Supplementary Materials) and Reference 2 (Tables S3 and S4, Figure S2, Supplementary Materials).

## 4. Conclusions

Metabolomics is a systems biology approach to understand human health and disease. It often involves identifying metabolite differences in biofluids collected from large numbers of healthy and diseased patients. Techniques that provide quantifiable, reproducible metabolite measurement, and that cover a broad dynamic range are essential. One-dimensional (1D) ^1^H-NMR is commonly used in metabolomics, but it suffers from low resolution. Two-dimensional (2D) NMR experiments significantly improve spectral resolution, but at the cost of longer experimental times or diminished sensitivity. Several landmark studies [35,36,37] demonstrated the feasibility of NUS spectroscopy to overcome many of these limitations.

Herein, we expanded upon these previous results using an increased number of metabolites and a broader range of concentrations to evaluate the utility of 2D ^1^H–^13^C NUS HSQC for metabolomics. We demonstrated that, using a 600-MHz NMR equipped with a cryoprobe, an NUS experiment was able to provide an HSQC spectrum in one-fourth of the time with little loss of information. All of the metabolites in standard samples were readily identifiable, and all but weak peaks in a plasma sample were detectable. Importantly, this was accomplished without the inclusion of relaxation enhancement agents. We plan to investigate if the addition of Cu(EDTA) or other relaxation agents improves either the sensitivity or the reproducibility of NUS experiments. The NUS spectrum did contain more artefacts than the US spectrum, which requires careful analysis to avoid erroneous interpretation. Much like the systematic parameter optimization for homonuclear NUS experiments conducted by Schlippenbach [35], we are also currently exploring effects of sampling density, NUS schedule, reconstruction options, etc. to determine optimal conditions to reduce artefacts. NUS dramatically improved the ability to characterize macromolecular structures and dynamics by NMR, and we believe that it will add a similar value to metabolomics.

We also confirmed several analytical properties of NUS NMR for metabolomics. The linearity of NUS HSQC measurements, which was previously shown for only a handful of metabolites within a relative narrow concentration range [36], was further confirmed. Herein, we demonstrated that NUS linearity is maintained across different metabolite chemical classes, and it was extended from the µM to mM range. In total, our results combined with prior reports [35,36,37] validates the ability of NUS to detect concentration changes for a variety of metabolites across multiple samples. Finally, we rigorously tested the repeatability, stability, and reproducibility of NUS HSQC measurements. Knowing the uncertainty associated with any analytical platform is fundamental to assessing the accuracy of a metabolomics study and avoiding misinterpretations. For example, by using a peak intensity cut-off, we can estimate the level of variation in our studies (e.g., 2 × 10^6^ correlates with %CV < 20%). In summation, our evaluation of the NUS HSQC experiment provides a set of useful guidelines to inform the optimal design of future NUS HSQC metabolomic studies.

## Figures and Tables

**Table 1:** Correlation coefficient (*R^2^*) for measured metabolite resonances.

Metabolite	Peak 1	Peak 2	Peak 3	Peak 4	Peak 5	Peak 6	Peak 7
UDP	1.00	1.00	0.99	0.99	0.99	0.99	0.99
Cytidine	0.80	1.00	0.99	1.00	1.00	1.00	
Fructose	0.99	1.00	0.99	1.00	1.00	1.00	
Ribose 5-phosphate	0.99	0.97	1.00	0.99	1.00	0.99	
NAD	0.99	1.00	1.00	1.00	1.00	1.00	0.99
NAD	1.00	0.99	0.99	1.00			
AMP	0.97	0.99	0.96	0.98	0.99		
Glucose	0.98	1.00	0.84	0.83	1.00		
Histidine	0.97	1.00	0.99	1.00	1.00		
2-Hydroxyglutaric acid	0.99	0.99	0.99	0.99			
GTP	0.99	0.99	1.00	0.99			
Leucine	1.00	1.00	1.00	0.98			
Acetylcholine	1.00	1.00	1.00				
Cysteine	1.00	1.00	0.97				
Glucosamine	0.94	0.99	1.00				
Lysine	1.00	1.00	1.00				
Malic acid	0.93	0.98	1.00				
Alanine	1.00	1.00					
Arginine	0.98	1.00					
Choline	1.00	1.00					
Glutamic acid	0.98	1.00					
Glutamine	1.00	1.00					
Lactic acid	1.00	0.95					
Ornithine	0.93	1.00					
Citrate	0.93						
Acetyl-phosphate	1.00						
Fumaric acid	0.99						
Pyruvic acid	0.99						
Succinic acid	1.00						

Peak1 through Peak7 refer to the measured resonances for each molecule.

**Table 2:** Limit of detection (LOD) for measured metabolite resonances.

Metabolite	Peak 1	Peak 2	Peak 3	Peak 4	Peak 5	Peak 6	Peak 7	Minimal Conc. (mM)
UDP	0.013	0.021	0.022	0.024	0.016	0.021	0.026	0.013
Cytidine	0.033	0.018	0.019	0.016	0.024	0.023		0.016
Fructose	0.035	0.028	0.044	0.056	0.026	0.026		0.026
Ribose 5-phosphate	0.065	0.049	0.037	0.048	0.019	0.041		0.019
NAD	0.034	0.018	0.024	0.013	0.020	0.021	0.022	0.013
NAD	0.024	0.024	0.029	0.020				0.020
AMP	0.013	0.018	0.029	0.022	0.025			0.013
Glucose	0.042	0.047	0.047	0.093	0.037			0.037
Histidine	0.036	0.034	0.022	0.022	0.027			0.022
2-Hydroxyglutaric acid	0.039	0.046	0.032	0.022				0.022
GTP	0.033	0.021	0.023	0.026				0.021
Leucine	0.010	0.009	0.040	0.028				0.009
Acetylcholine	0.025	0.016	0.017					0.016
Cysteine	0.058	0.038	0.047					0.038
Glucosamine	0.058	0.048	0.028					0.028
Lysine	0.048	0.021	0.019					0.019
Malic acid	0.079	0.066	0.020					0.020
Alanine	0.012	0.058						0.012
Arginine	0.044	0.011						0.011
Choline	0.019	0.023						0.019
Glutamic acid	0.042	0.020						0.020
Glutamine	0.020	0.019						0.019
Lactic acid	0.011	0.067						0.011
Ornithine	0.069	0.013						0.013
Acetyl-phosphate	0.024							0.024
Citrate	0.014							0.014
Fumaric acid	0.025							0.025
Pyruvic acid	0.019							0.019
Succinic acid	0.009							0.009

Peak1 through Peak7 refer to the measured resonances for each molecule.

**Table 3:** Limit of quantification (LOQ) for measured metabolite resonances.

Metabolite	Peak 1	Peak 2	Peak 3	Peak 4	Peak 5	Peak 6	Peak 7	Minimal Conc. (mM)
UDP	0.044	0.071	0.075	0.081	0.055	0.068	0.087	0.044
Cytidine	0.111	0.061	0.064	0.054	0.081	0.076		0.054
Fructose	0.117	0.094	0.148	0.187	0.086	0.086		0.086
Ribose 5-phosphate	0.216	0.162	0.123	0.159	0.064	0.138		0.064
NAD	0.112	0.059	0.081	0.044	0.067	0.071	0.075	0.044
NAD	0.079	0.080	0.097	0.066				0.066
AMP	0.044	0.059	0.098	0.073	0.083			0.044
Glucose	0.139	0.156	0.155	0.311	0.124			0.124
Histidine	0.121	0.113	0.074	0.075	0.092			0.074
2-Hydroxyglutaric acid	0.131	0.152	0.106	0.072				0.072
GTP	0.111	0.070	0.077	0.088				0.070
Leucine	0.032	0.031	0.133	0.092				0.031
Acetylcholine	0.082	0.054	0.056					0.054
Cysteine	0.194	0.126	0.158					0.126
Glucosamine	0.194	0.160	0.094					0.094
Lysine	0.161	0.069	0.064					0.064
Malic acid	0.265	0.222	0.066					0.066
Alanine	0.039	0.194						0.039
Arginine	0.147	0.037						0.037
Choline	0.065	0.077						0.065
Glutamic acid	0.139	0.068						0.068
Glutamine	0.066	0.064						0.064
Lactic acid	0.038	0.223						0.038
Ornithine	0.229	0.045						0.045
Acetylphosphate	0.081							0.081
Citrate	0.048							0.048
Fumaric acid	0.084							0.084
Pyruvic acid	0.063							0.063
Succinic acid	0.087							0.087

Peak1 through Peak7 refer to the measured resonances for each molecule.

## Supplementary Materials

The following are available online at https://www.mdpi.com/2218-1989/10/5/203/s1: Figure S1. 2D ^13^C–^1^H HSQC spectrum of Reference 1 (see Table S1 for metabolite list and Table S2 for assignments); Figure S2. 2D ^13^C–^1^H HSQC spectrum of Reference 2 (see Table S3 for metabolite list and Table S4 for assignments); Figure S3. US vs. 25% NUS; Figure S4. Linear regression curve of Reference 2; Figure S5. Probe sensitivity contributes to variation. Comparison of coefficient of variation (CV) Reference 1 at 0.5 mM collected on a TXI (blue) and TCI (orange) probe; Table S1. The list of metabolites in Reference 1 model mixture; Table S2: Peak assignment for 15 metabolites in Reference 1; Table S3. The list of metabolites in Reference 2 model mixture; Table S4. Peak assignment for 29 metabolites in Reference 2; Table S5. The peaks that were used to calculate *R^2^*, LOD, and LOQ; Table S6. The peaks that were missing in 50% and 25% NUS compared to the US spectrum.
