# Supplementary material for: Evaluation of Non-Uniform Sampling 2D 1H–13C HSQC Spectra for Semi-Quantitative Metabolomics
Source: Metabolites. 2020 May 16;10(5):203. doi: 10.3390/metabo10050203 (PMC7281502; doi:10.3390/metabo10050203)
Supplement: Supplementary file 1 [file metabolites-10-00203-s001.pdf]

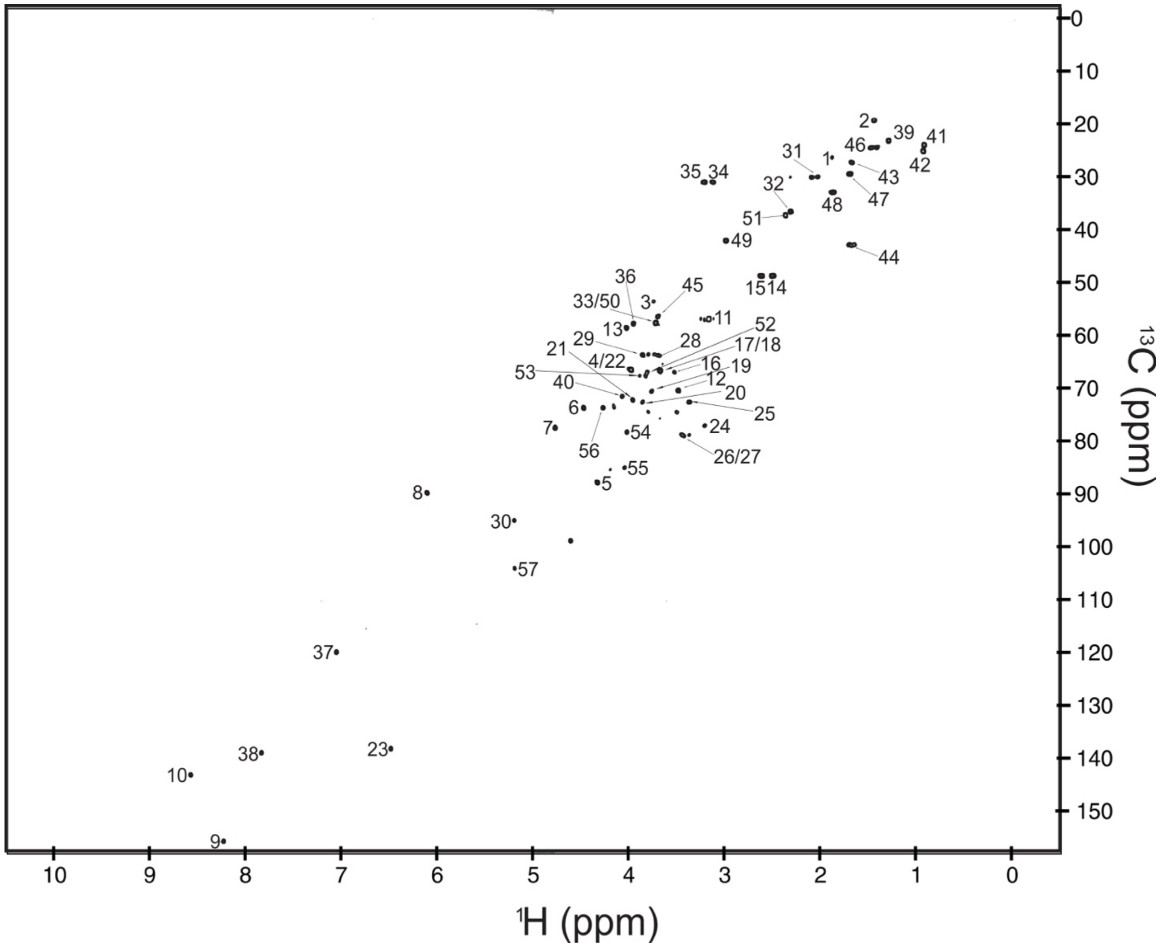

Figure S1. 2D ^{13}C-^{1}H HSQC spectrum of Reference 1 (see Table S1 for metabolite list and Table S2 for assignments).

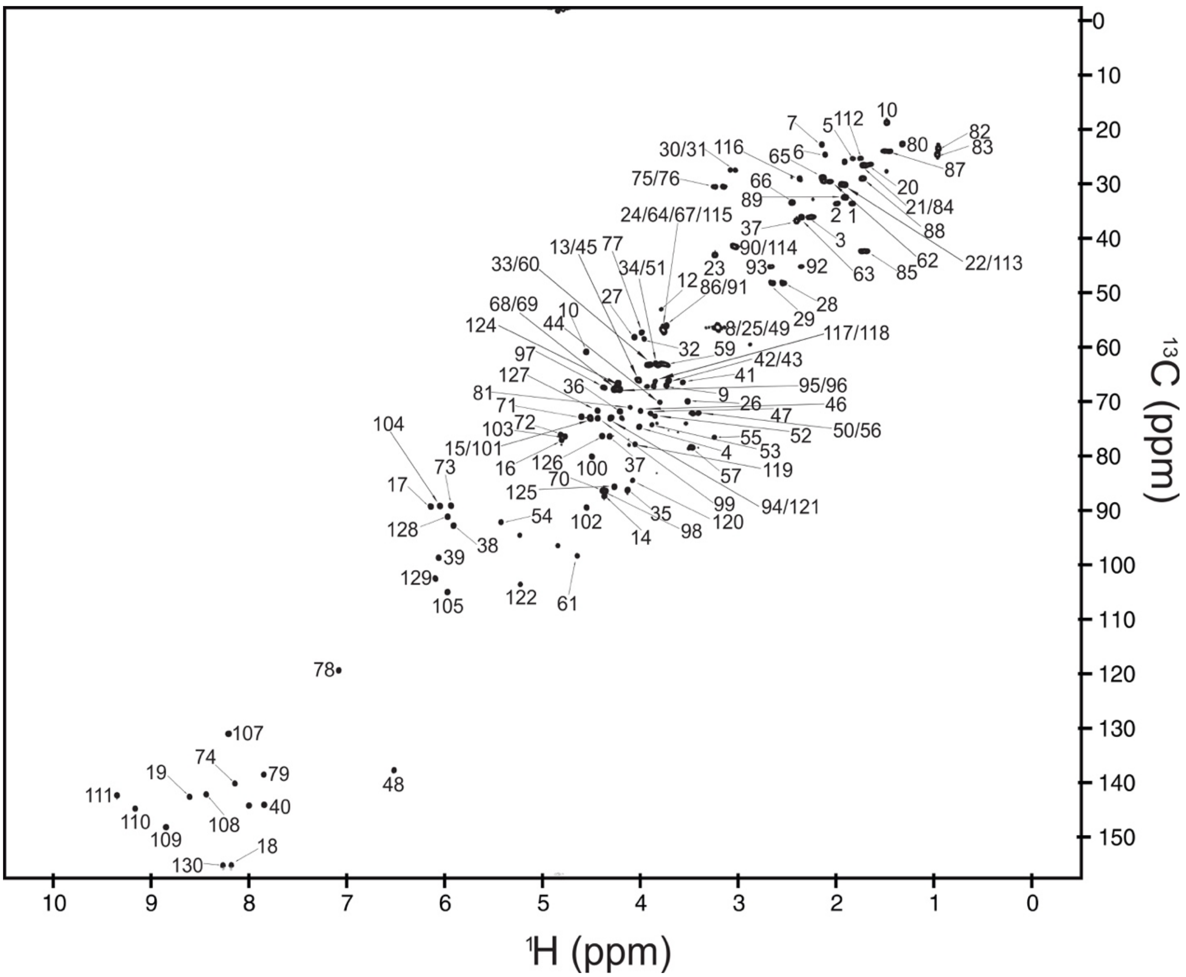

Figure S2. 2D ^{13}C-^{1}H HSQC spectrum of Reference 2 (see see Table S3 for metabolite list and Table S4 for assignments).

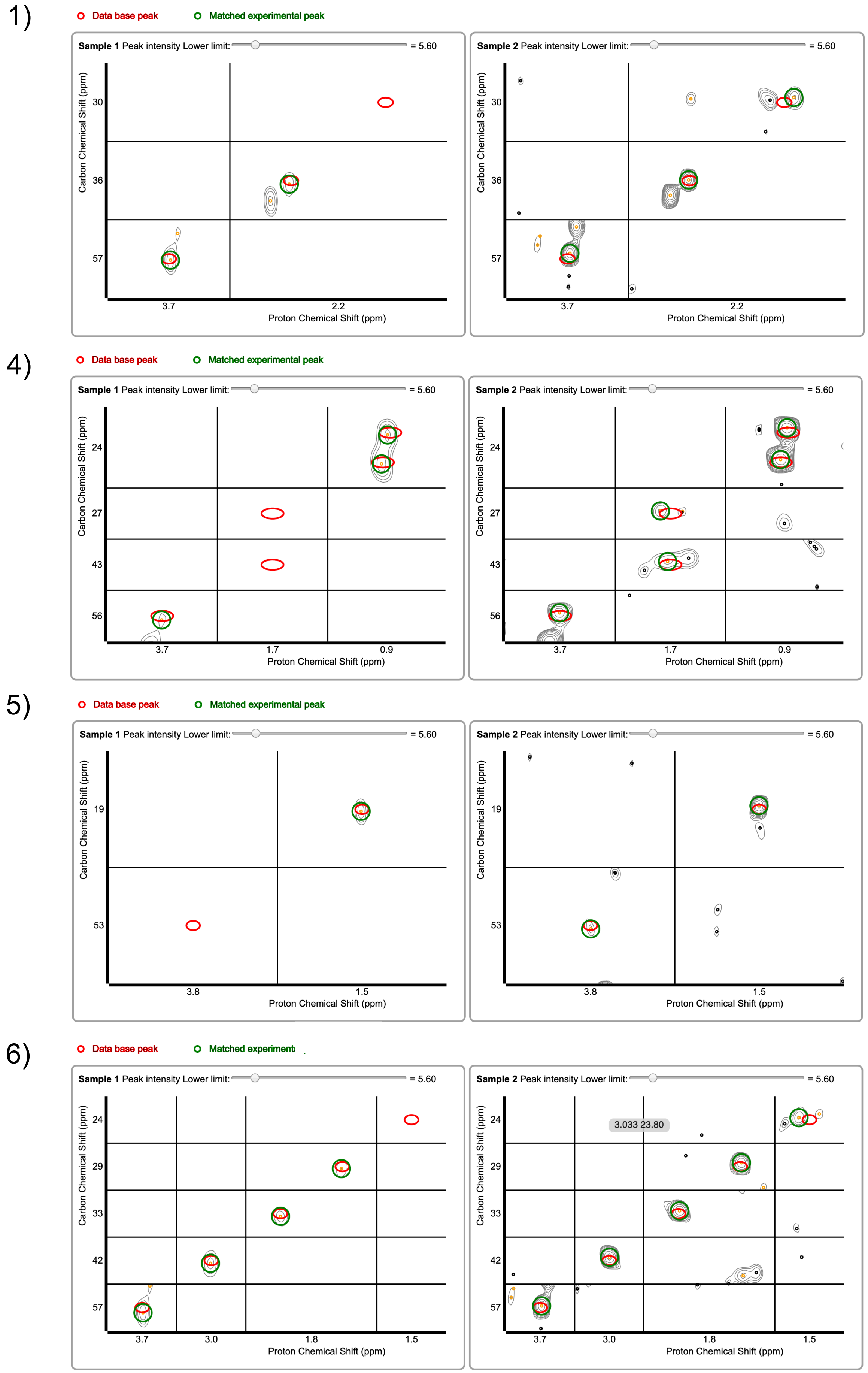

Figure S3. US vs 25% NUS: 1) Glutamic acid 4) Leucine 5) Alanine 6) Lysine

9)

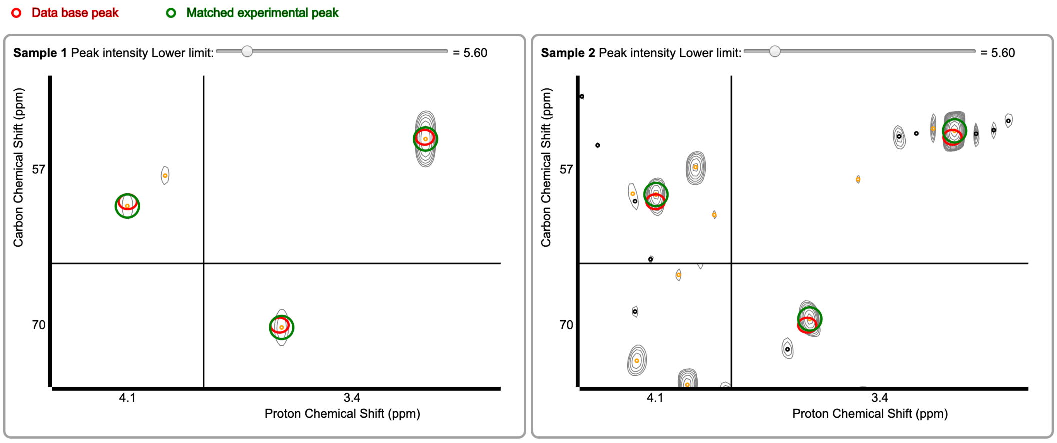

12)

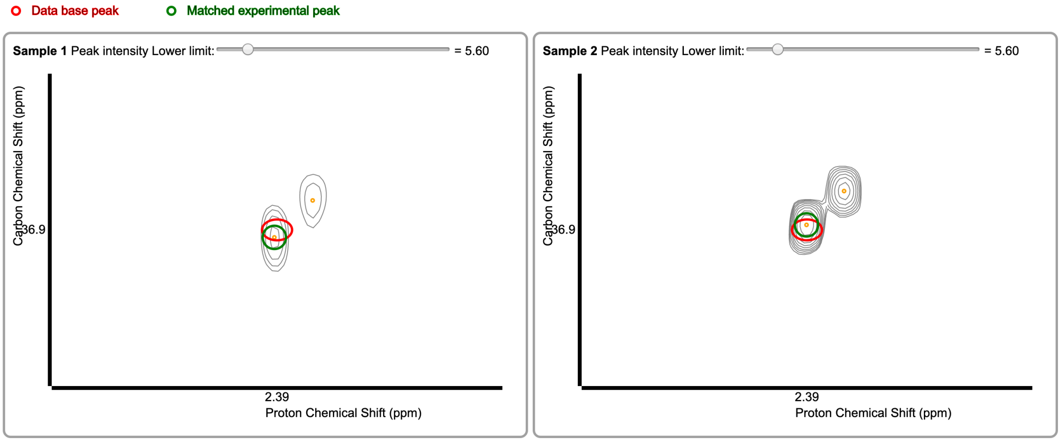

13)

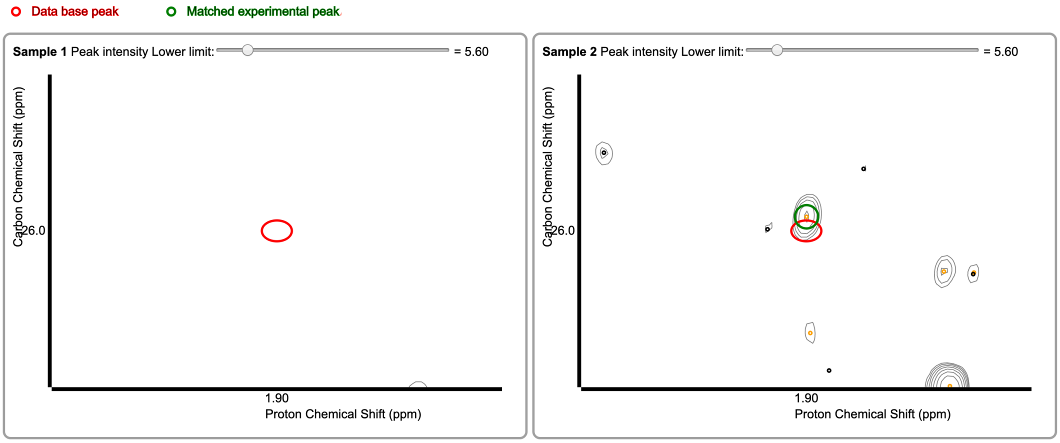

14)

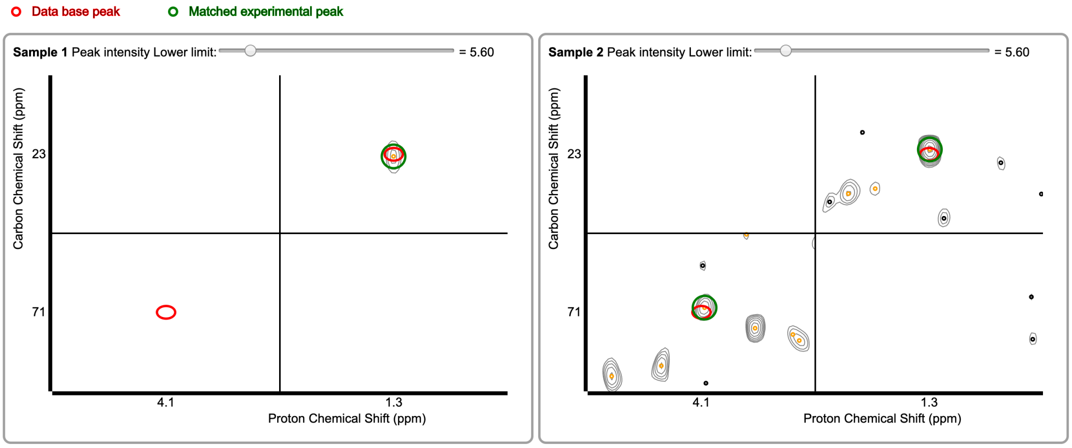

Figure S3 US vs 25% NUS (continued): 9) Choline 12) Succinic acid 13) Acetic acid 14) Lactic acid

16)

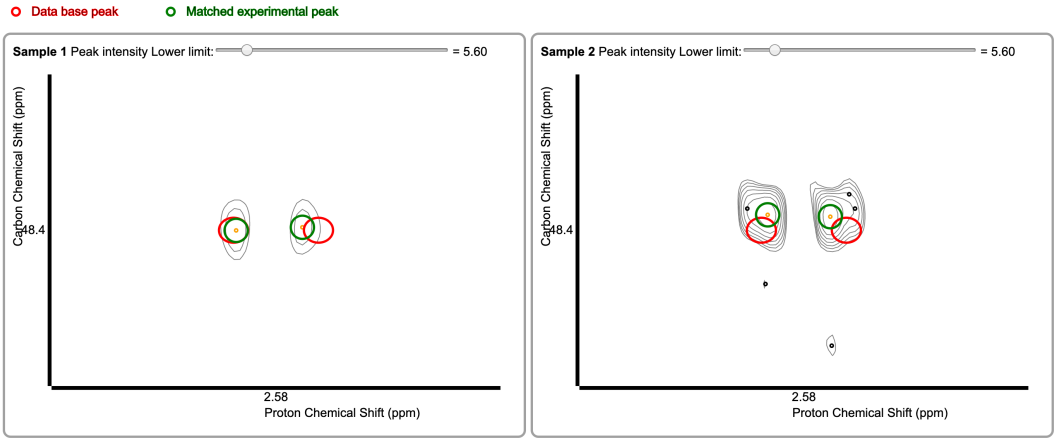

19)

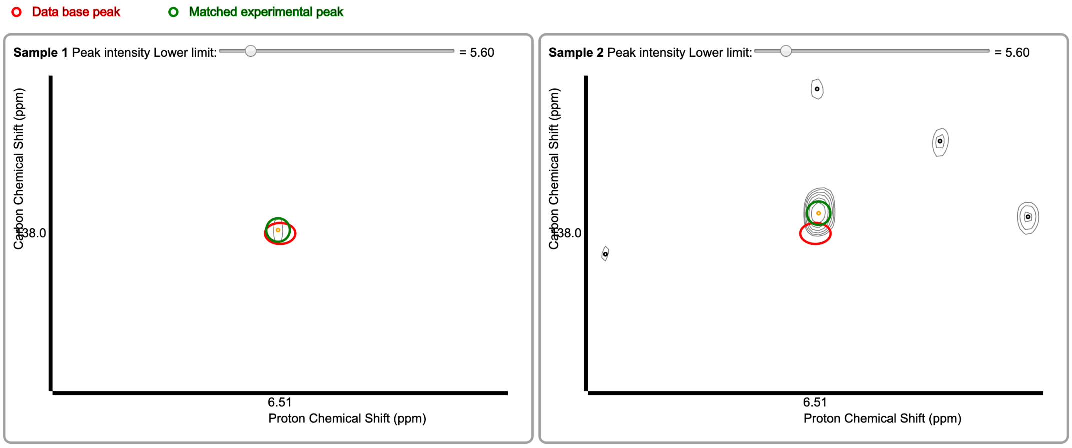

21)

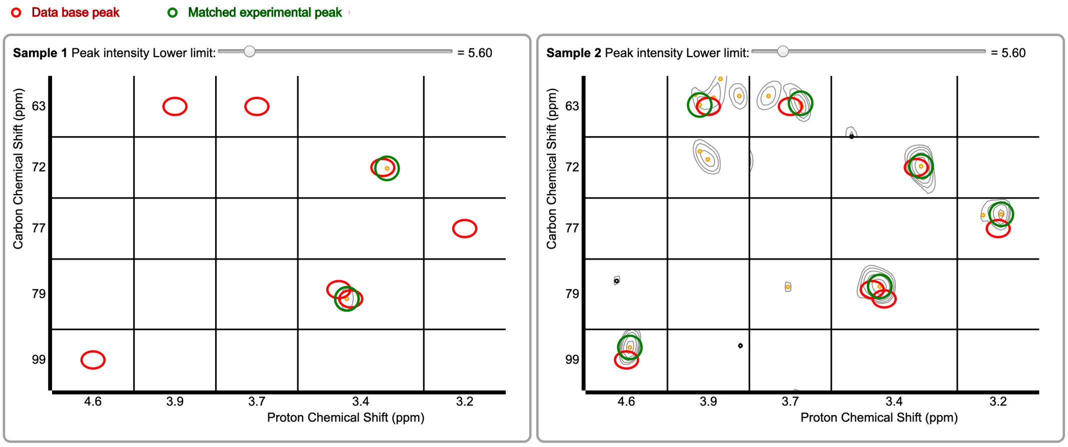

22)

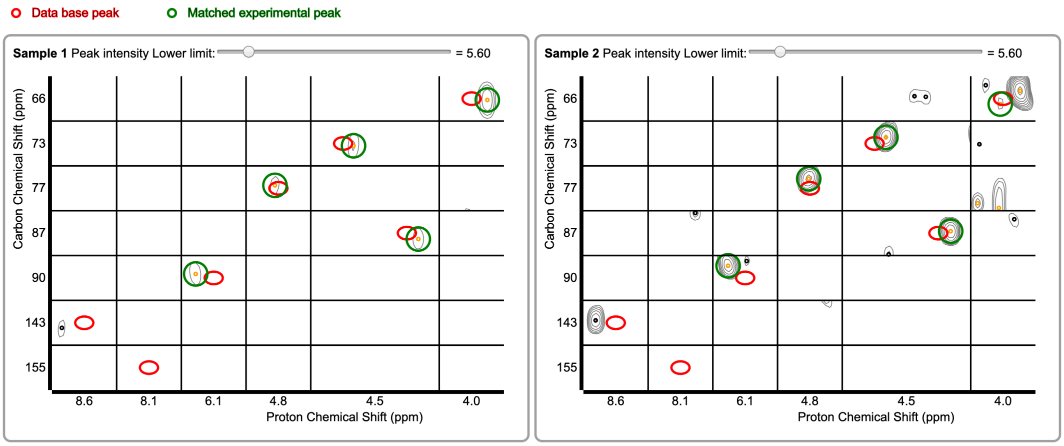

Figure S3 US vs 25% NUS (continued): 16) Citric acid 19) Fumaric acid 21) Glucose 22) AMP

27)

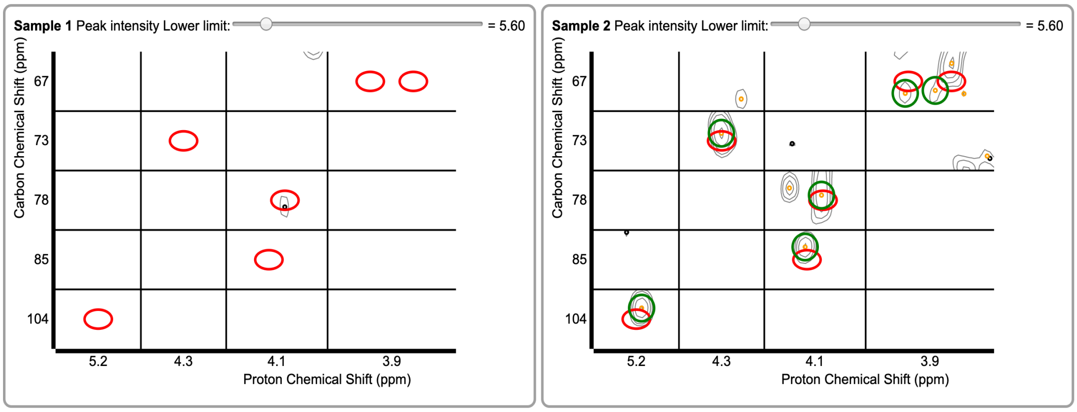

28)

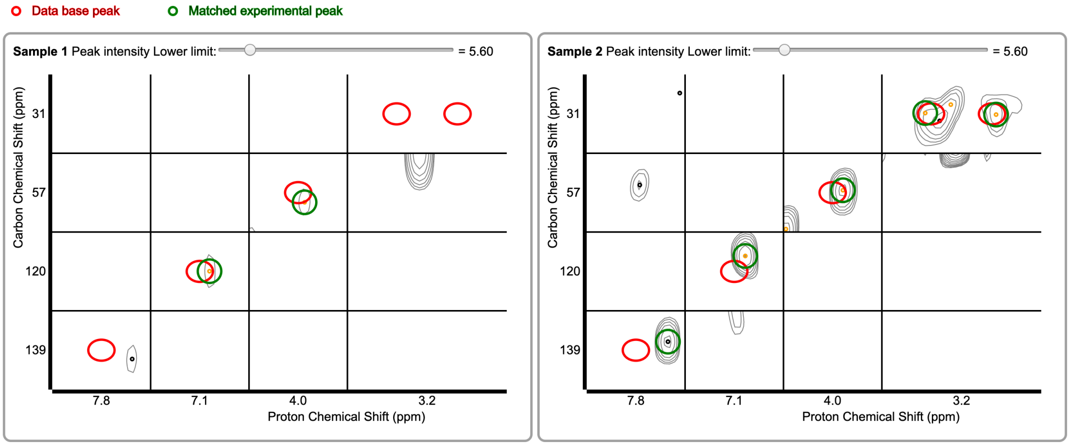

30)

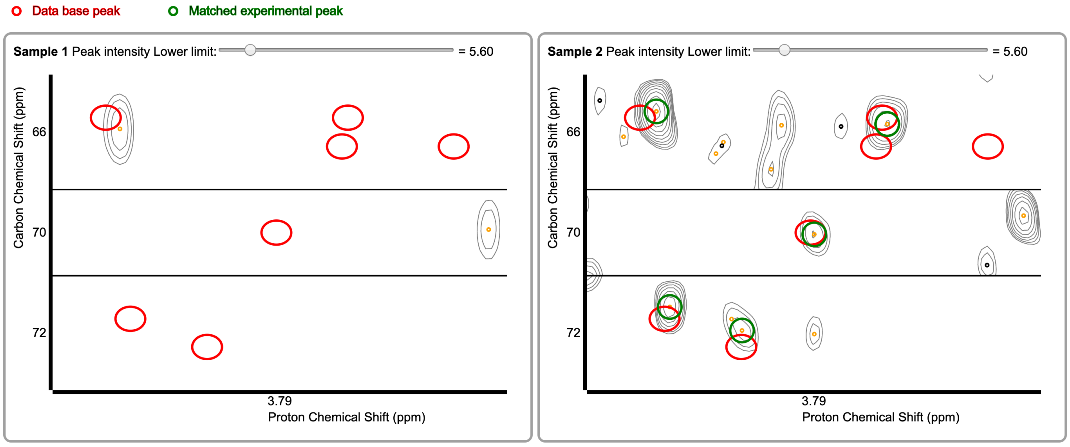

Figure S3 US vs 25% NUS (continued): 27) Ribose 5-phosphate 28) Histidine 30) Fructose

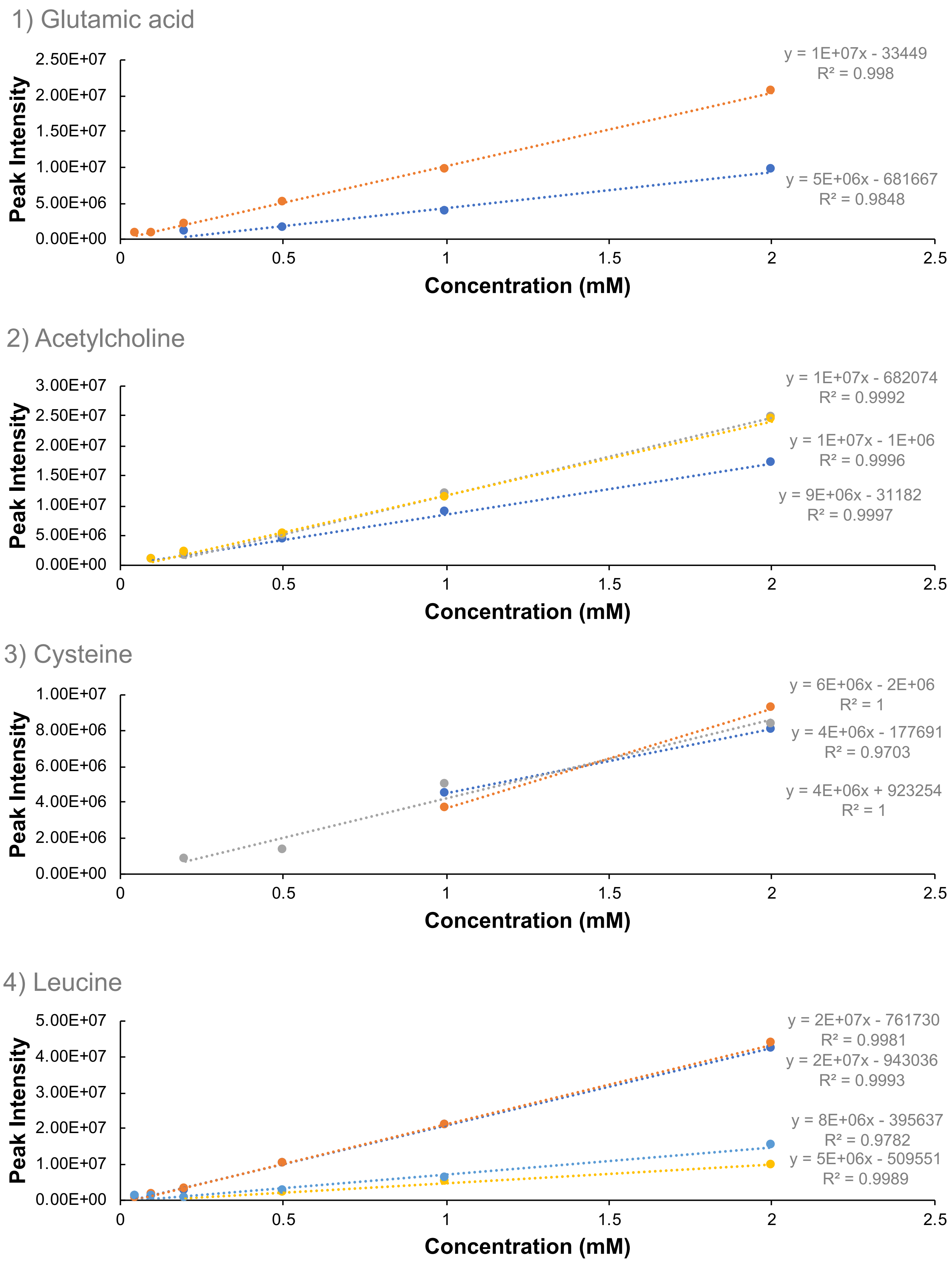

Figure S4. Linear regression curve of Reference 2

5) Alanine

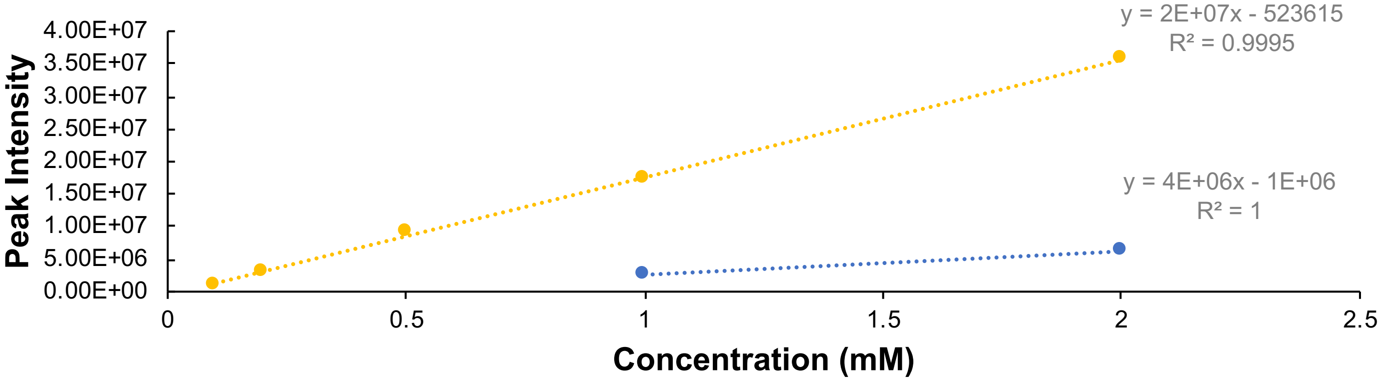

6) Lysine

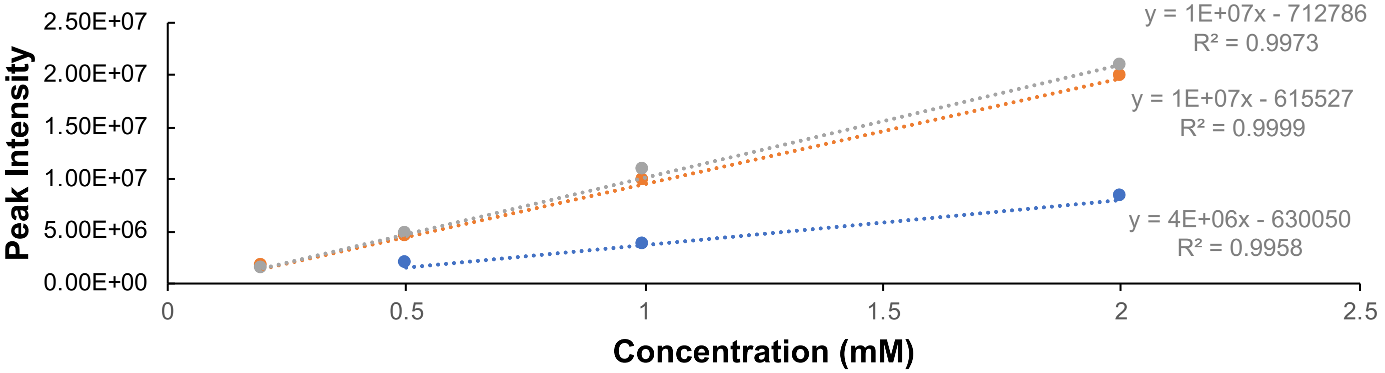

7) Ornithine

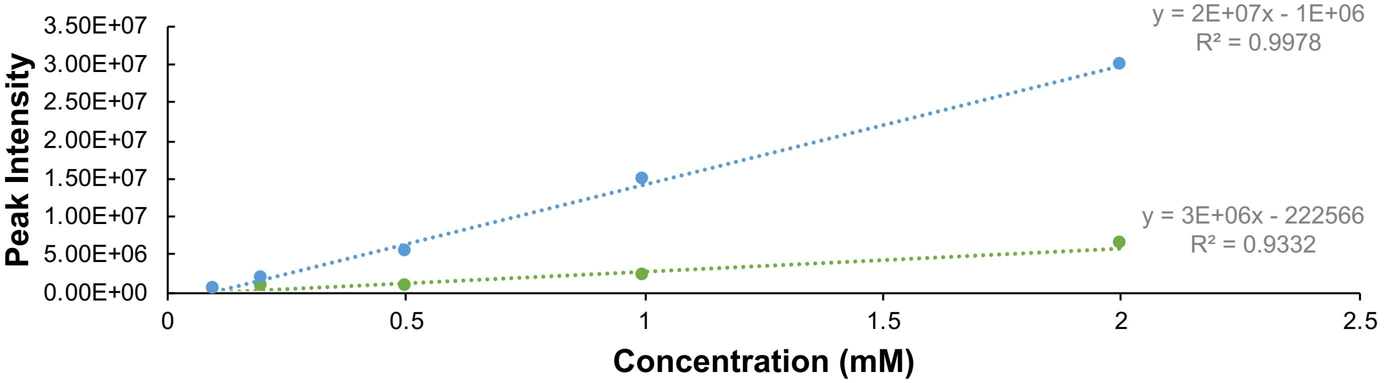

8) Arginine

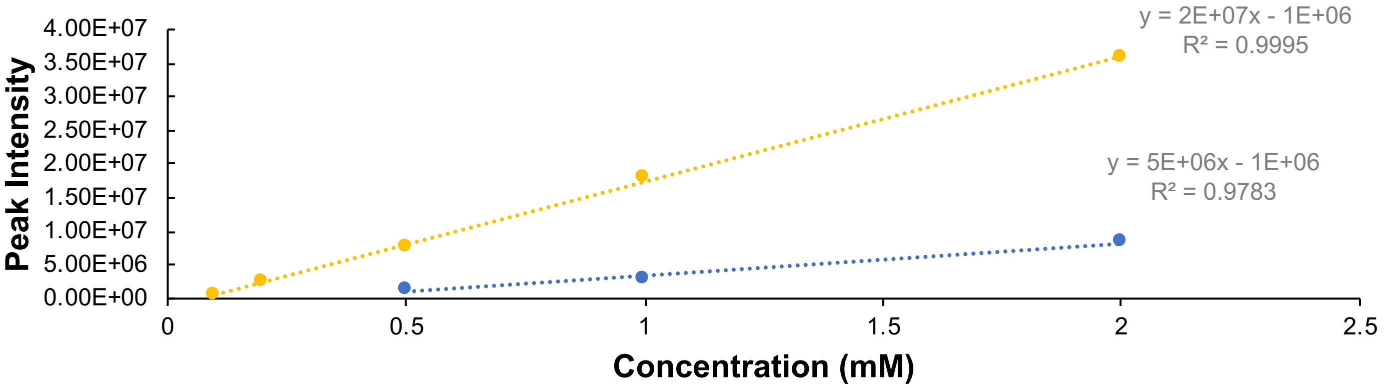

Figure S4 (continued). Linear regression curve of Reference 2

9) Choline

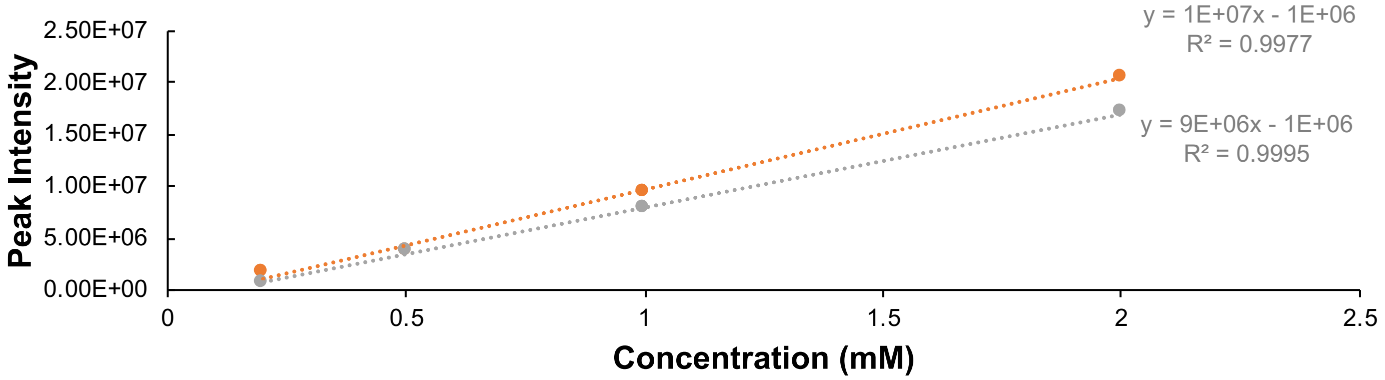

10) Glutamine

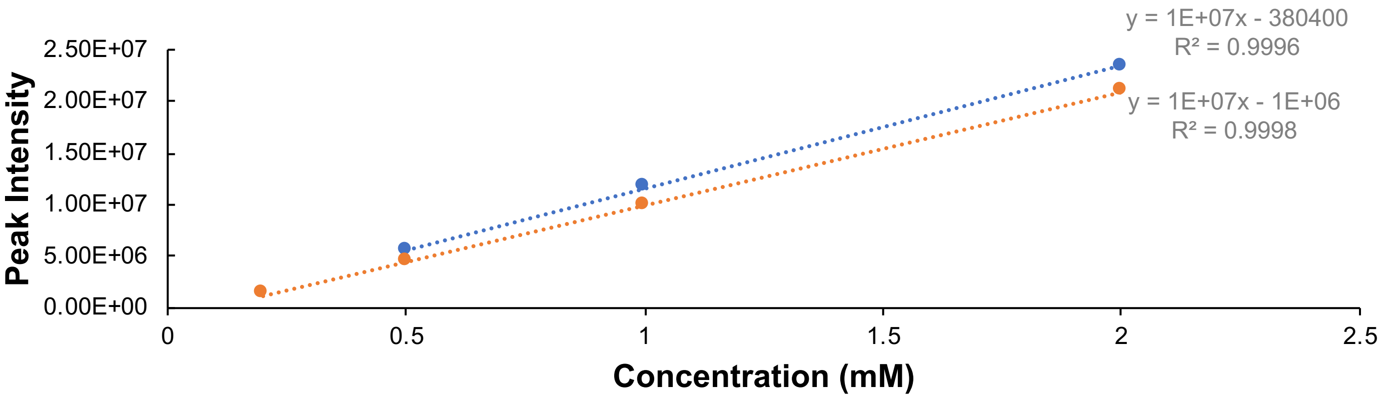

11) Succinic acid

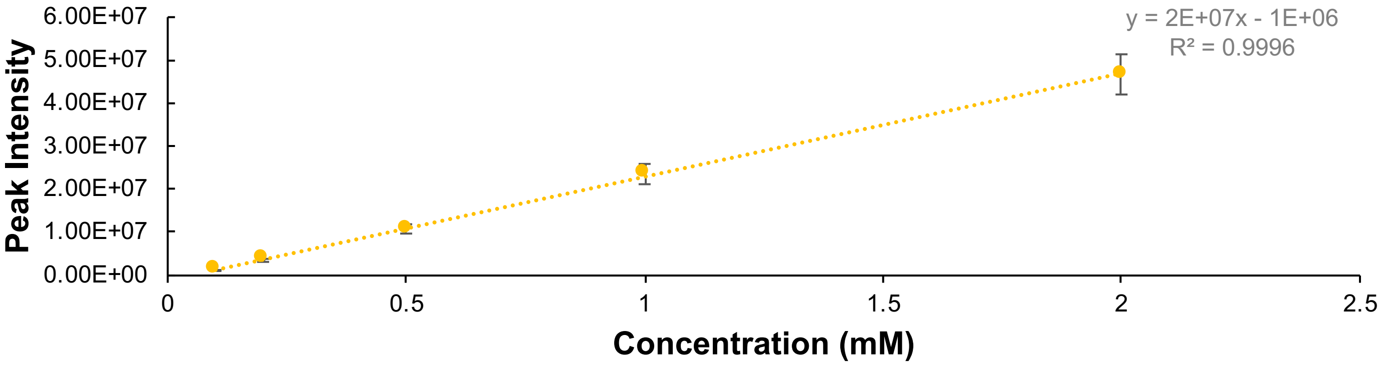

12) Acetic acid

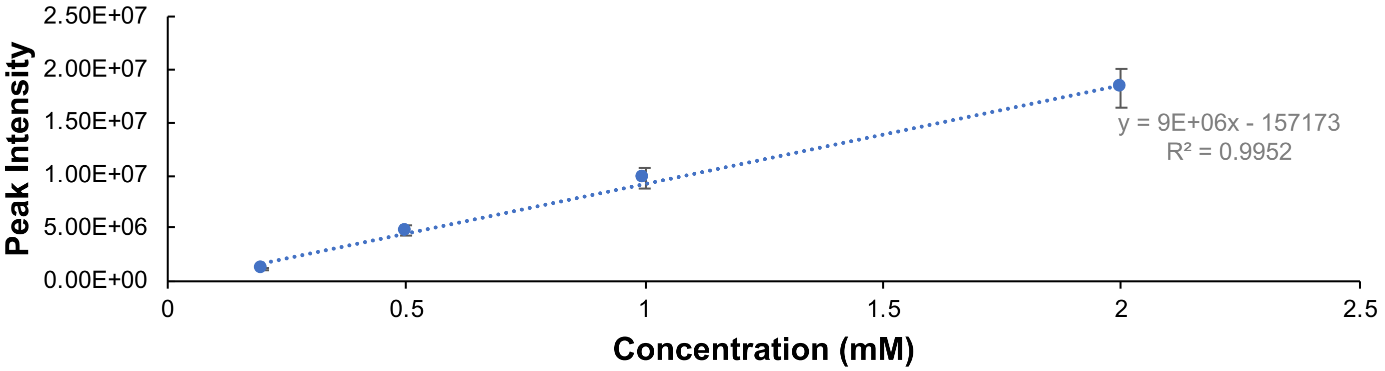

Figure S4 (continued). Linear regression curve of Reference 2

13) Lactic acid

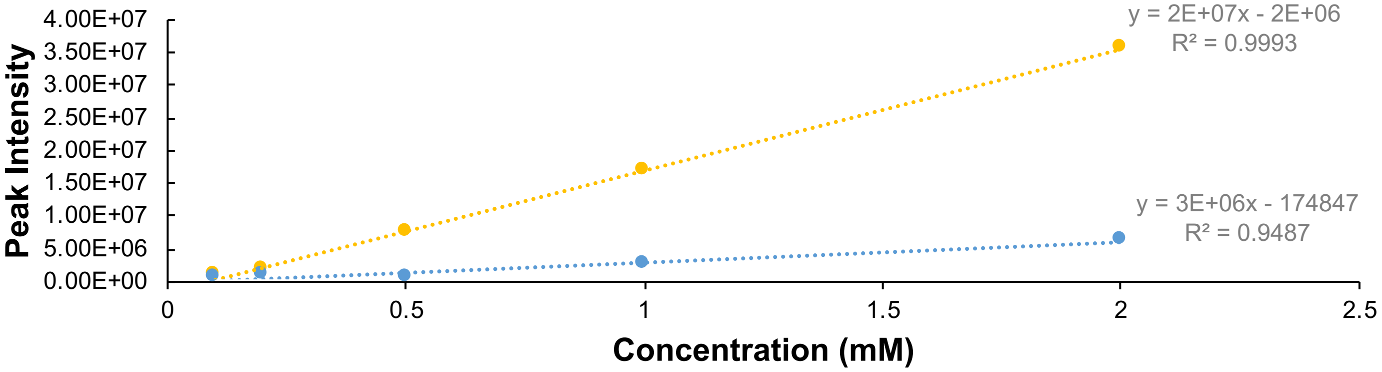

14) Malic acid

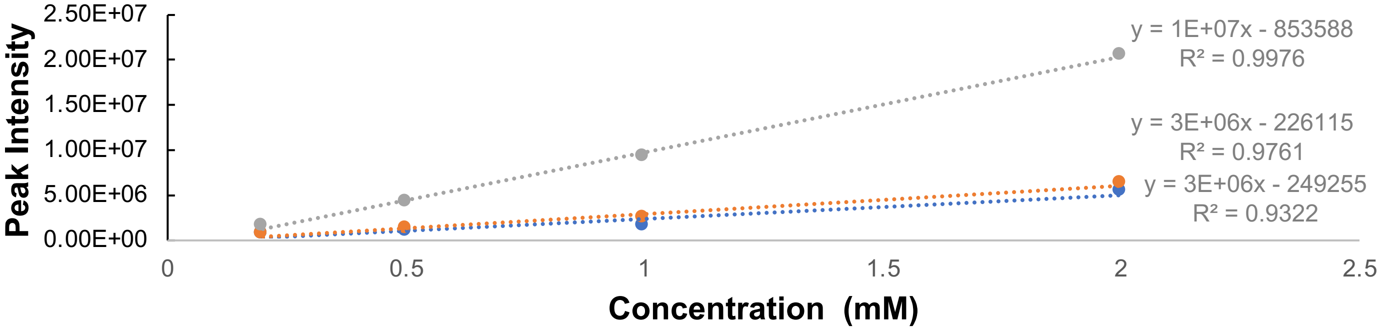

15) Citric acid

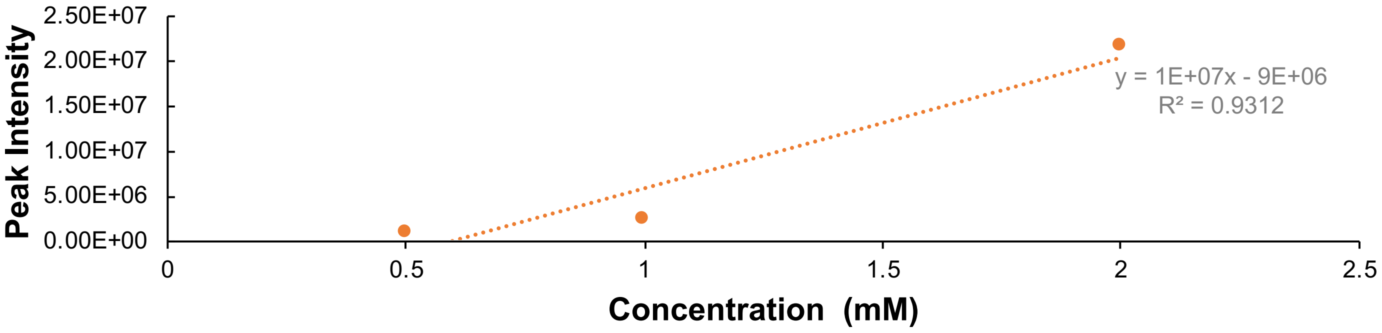

16) Pyruvic acid

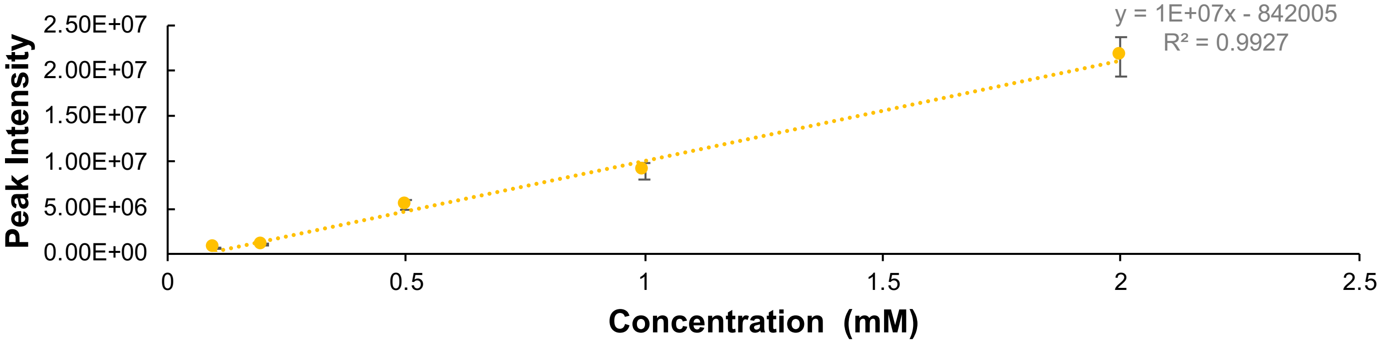

Figure S4 (continued). Linear regression curve of Reference 2

17) Acetylphosphate

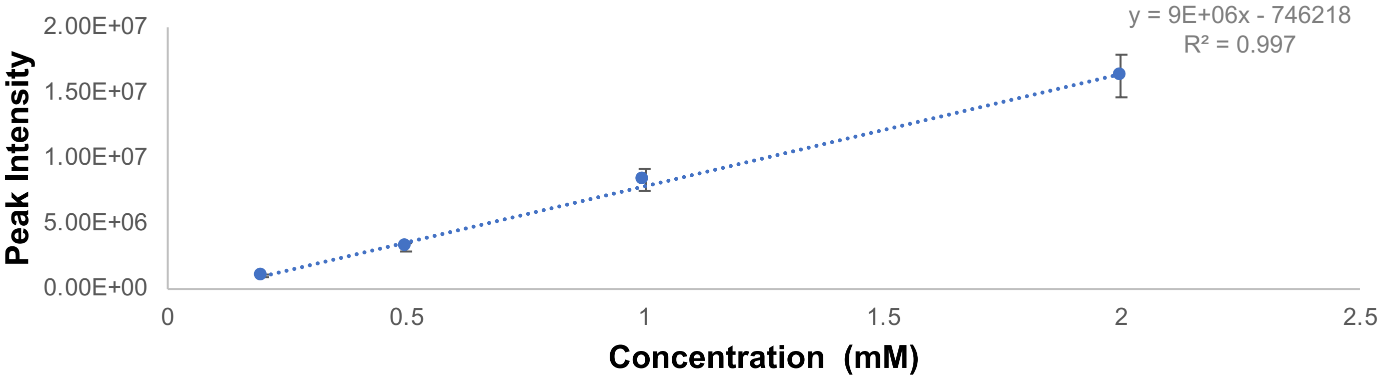

18) Fumaric acid

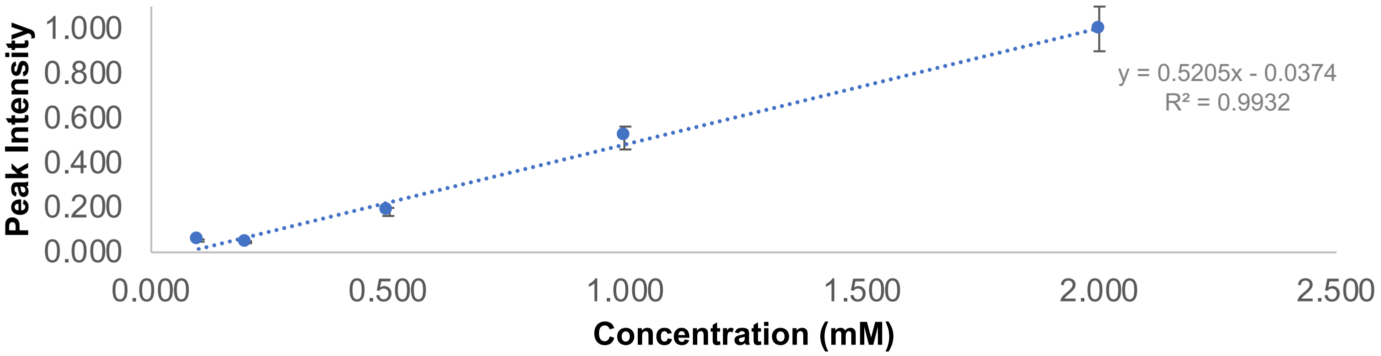

19) 2-Hydroxyglutaric acid

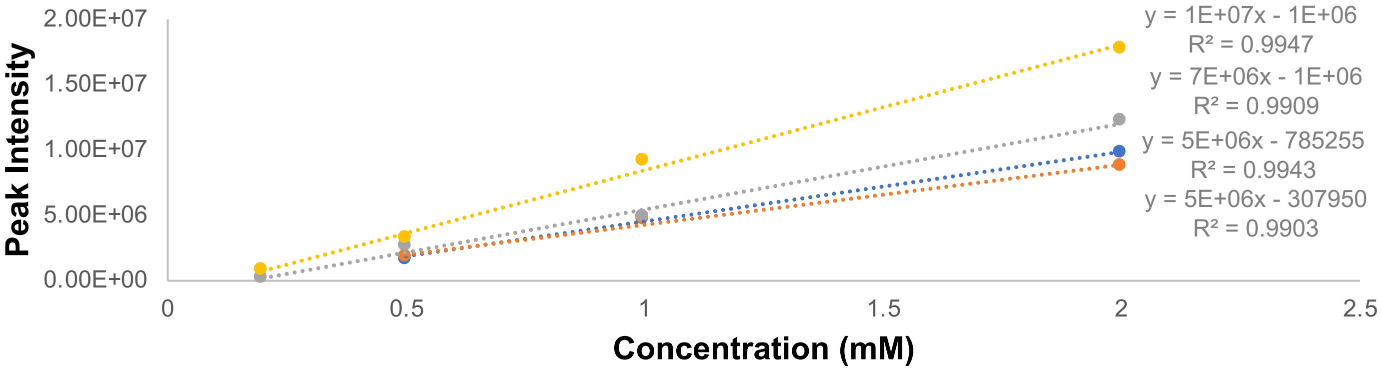

20) Glucose

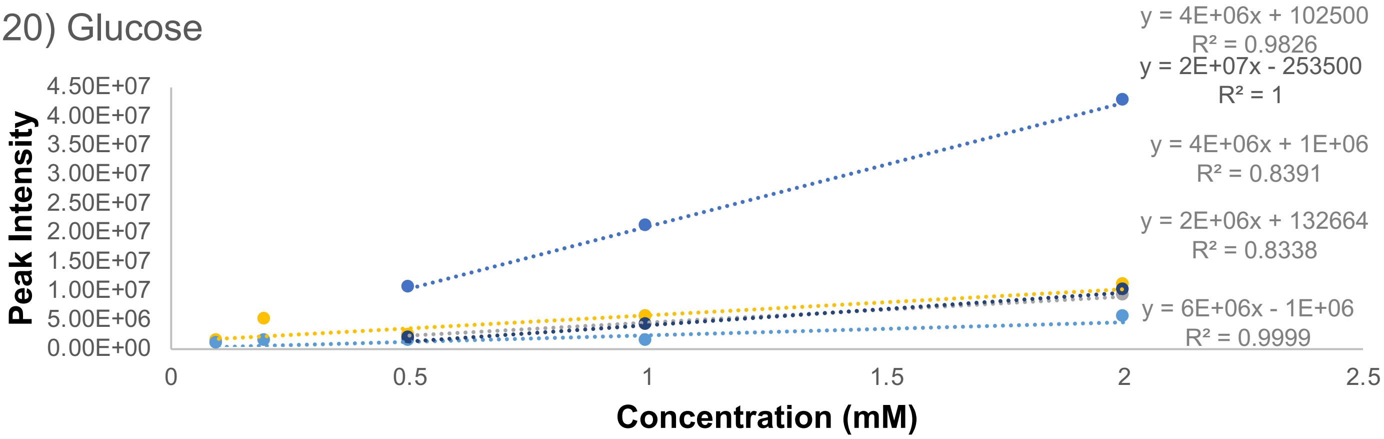

Figure S4 (continued). Linear regression curve of Reference 2

21) AMP

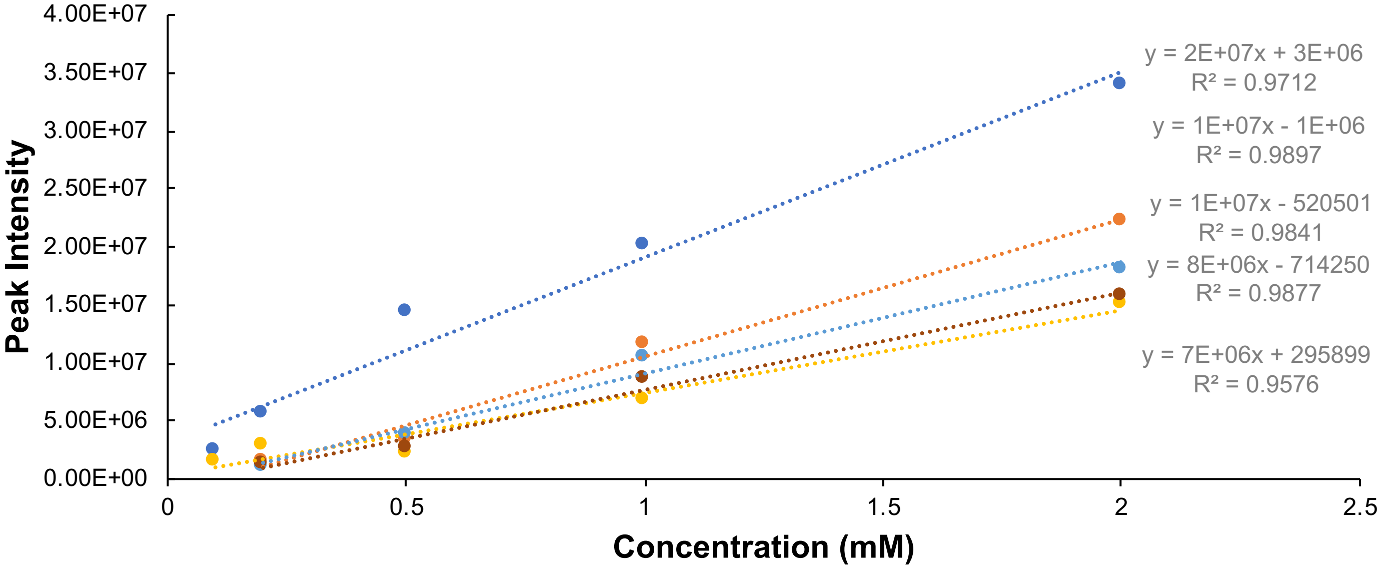

22) NAD

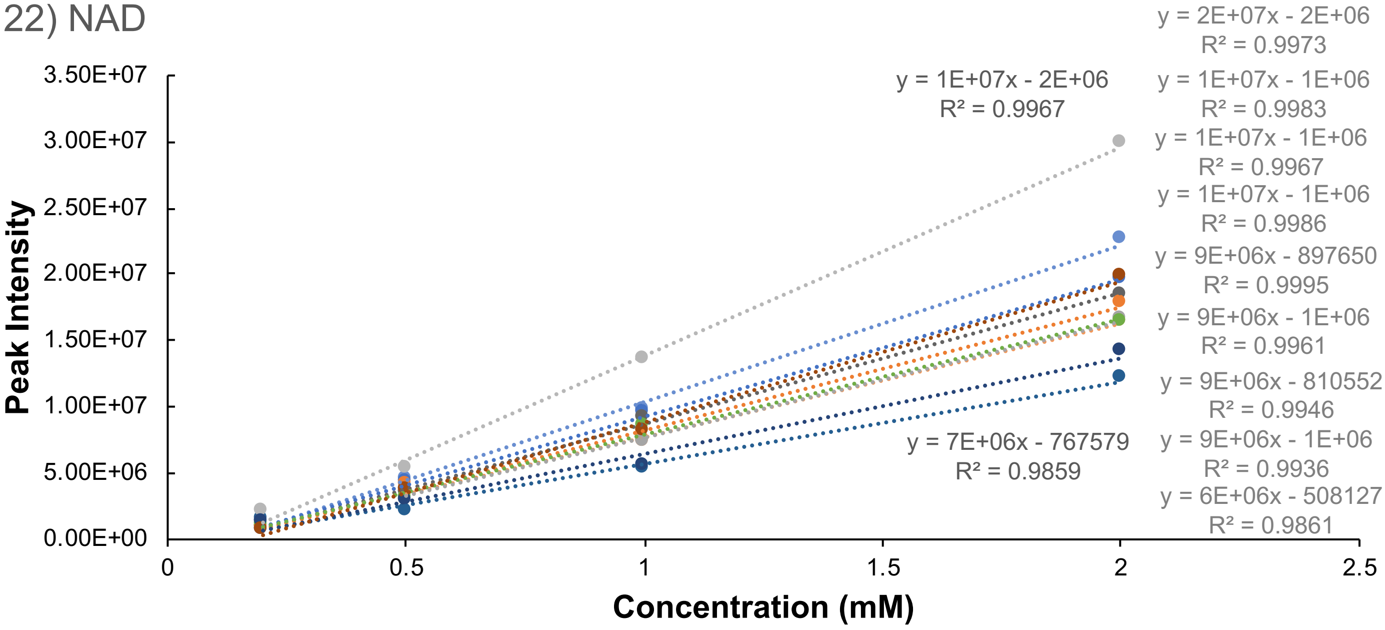

23) Glucosamine

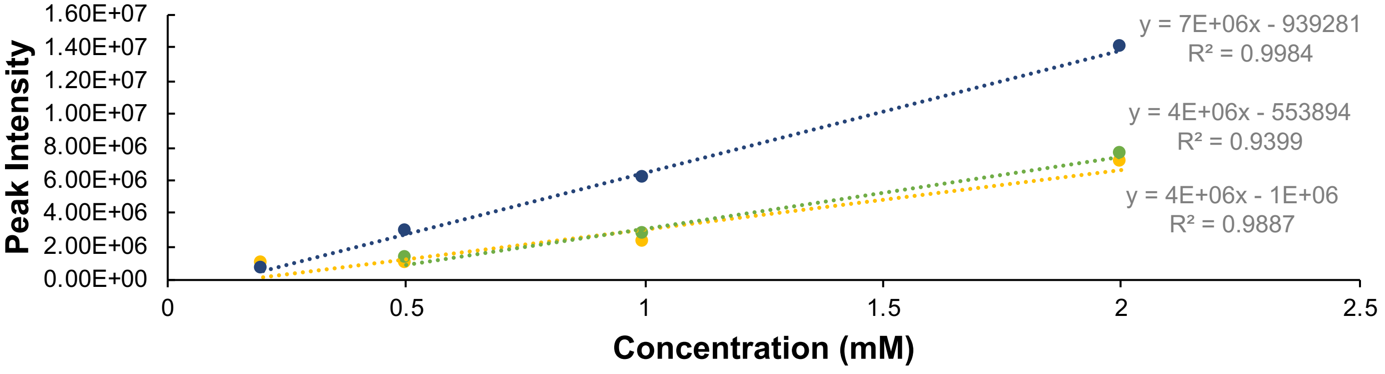

Figure S4 (continued). Linear regression curve of Reference 2

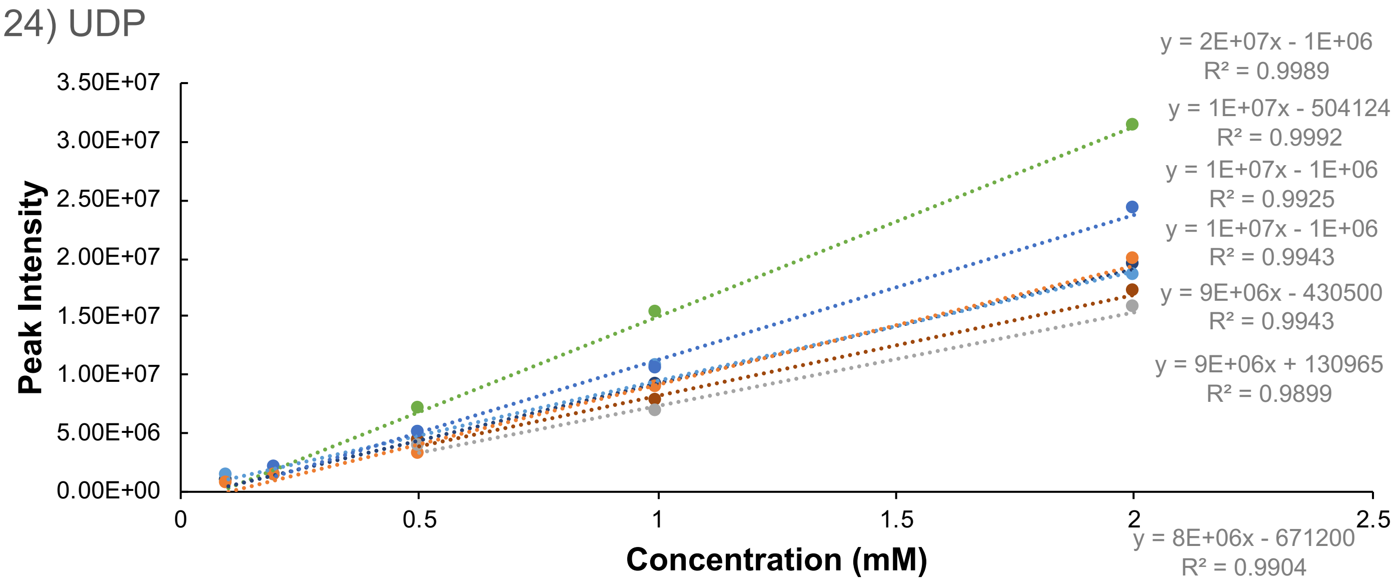

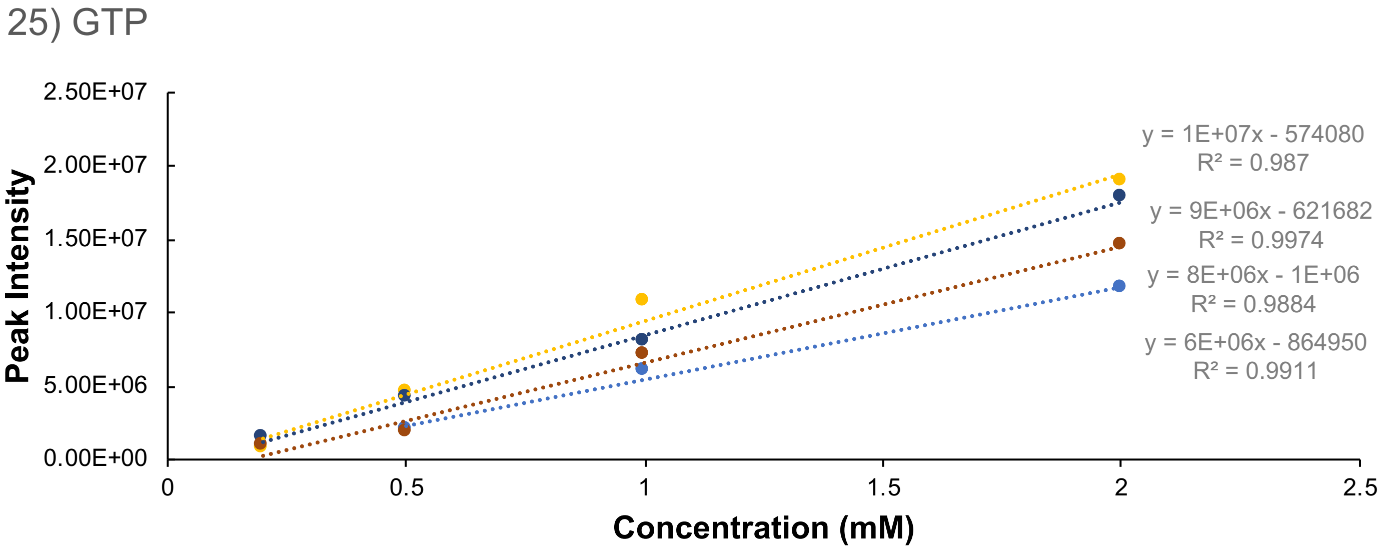

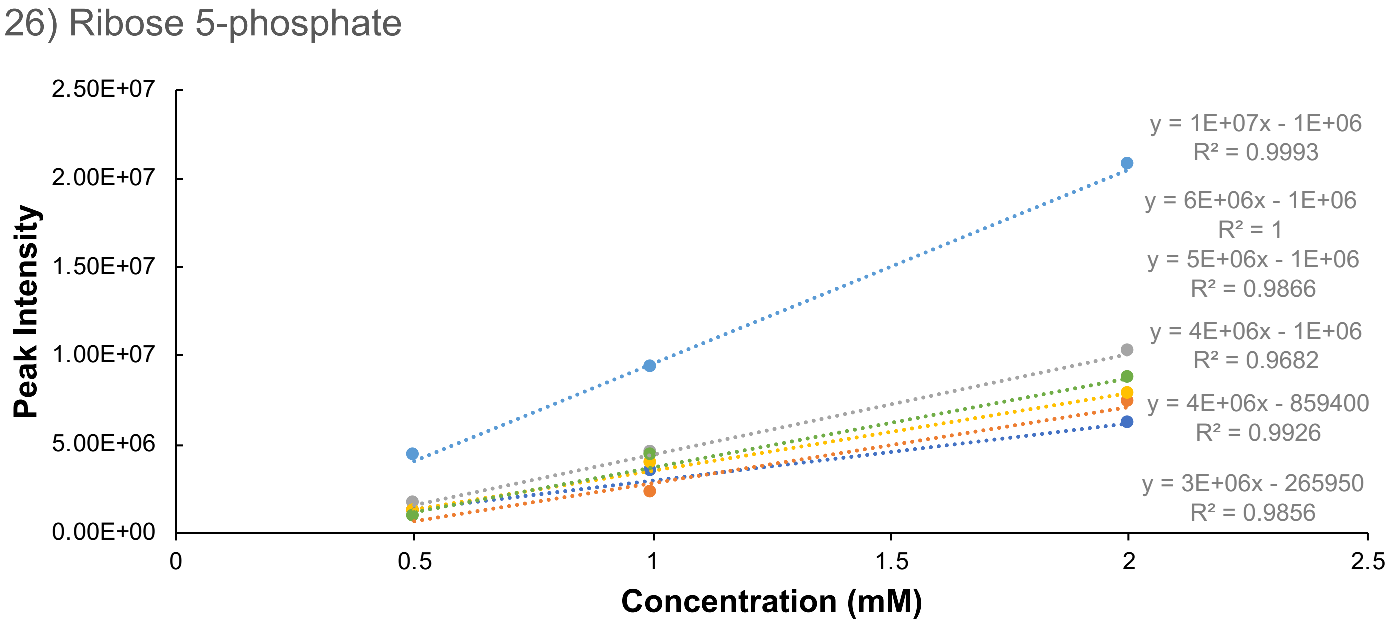

Figure S4 (continued). Linear regression curve of Reference 2

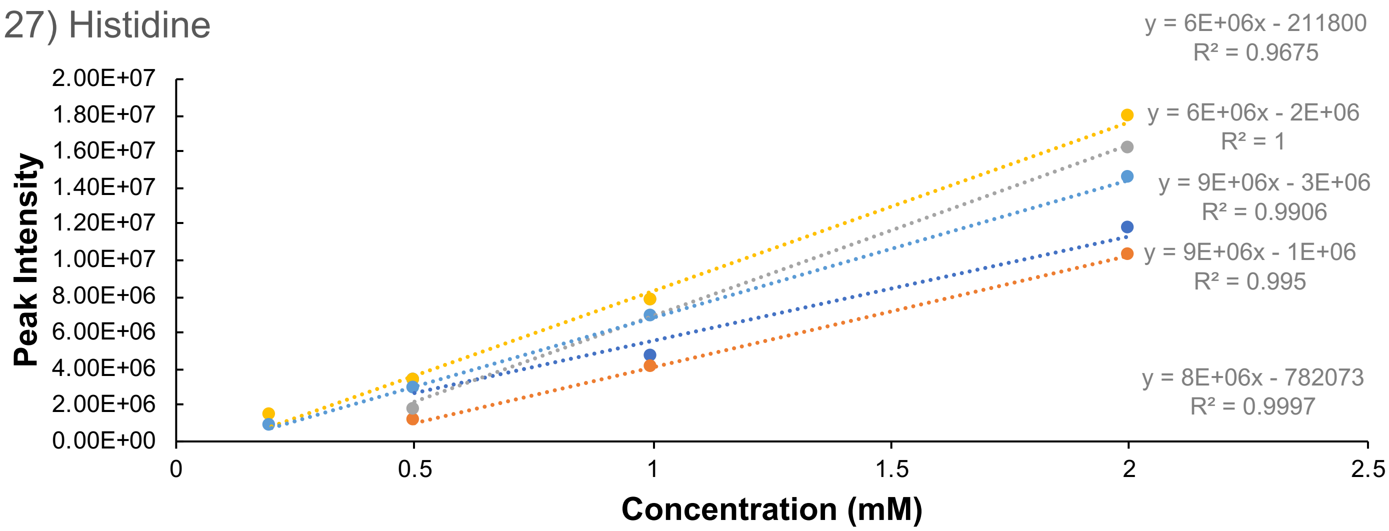

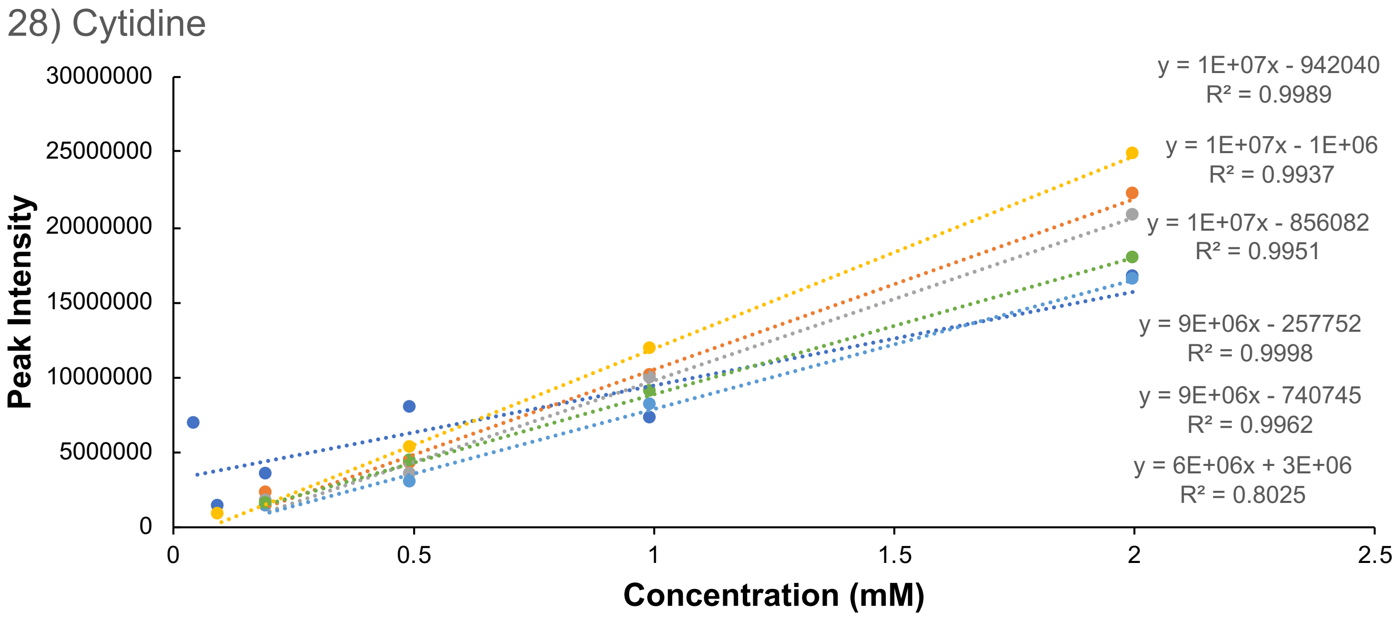

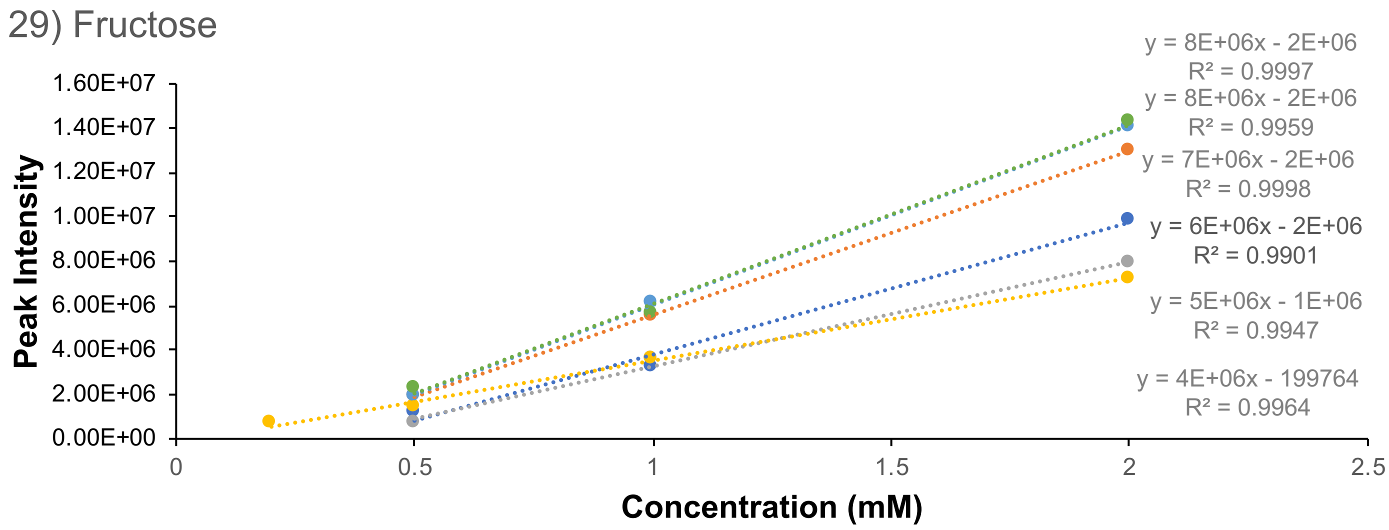

Figure S4 (continued). Linear regression curve of Reference 2

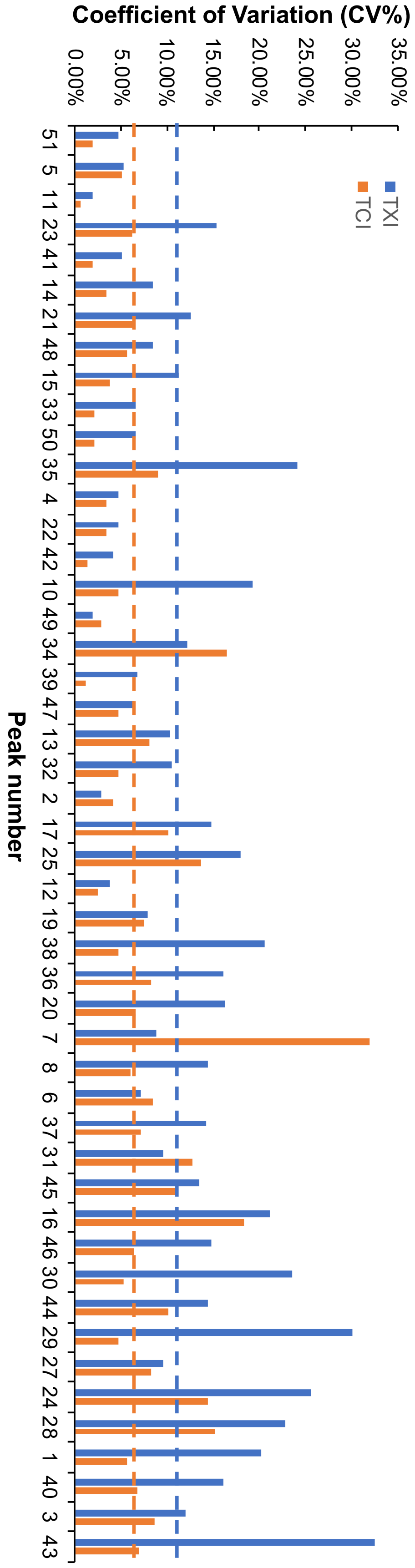

Figure S5. Probe Sensitivity Contributes to Variation. Comparison of coefficient of variation (CV) Reference 1 at 0.5 mM collected on a TXI (blue) and TCI (orange) probe.

Table S1. The list of metabolites in reference 1 model mixture.

No.	Metabolites	Concentration (μM)
1	Glutamic acid	500
4	Leucine	500
5	Alanine	500
6	Lysine	500
9	Choline	500
12	Succinic acid	500
13	Acetic acid	500
14	Lactic acid	500
16	Citric acid	500
19	Fumaric acid	500
21	Glucose	500
22	AMP	500
27	Ribose 5P	500
28	Histidine	500
30	Fructose	500

Table S2. Peak assignment for 15 metabolites in reference 1.

Peak #	Metabolites	Index	H (ppm)	C (ppm)
1	Acetic acid	0	1.9	25.99
2	Alanine_1	0	1.47	18.87
3	Alanine_2	1	3.77	53.22
4	AMP_1	0	4.04	66.24
5	AMP_2	1	4.38	87.42
6	AMP_3	2	4.53	73.45
7	AMP_4	3	4.79	77.29
8	AMP_5	4	6.09	89.64
9	AMP_6	5	8.11	155.34
10	AMP_7	6	8.56	142.77
11	Choline_1	0	3.19	56.58
12	Choline_2	1	3.51	70.12
13	Choline_3	2	4.05	58.33
14	Citrate_1	0	2.52	48.45
15	Citrate_2	1	2.64	48.45
16	Fructose_1	0	3.56	66.57
17	Fructose_2	1	3.7	66.09
18	Fructose_3	2	3.71	66.57
19	Fructose_4	3	3.79	70.23
20	Fructose_5	4	3.89	72.39
21	Fructose_6	5	3.99	71.93
22	Fructose_7	6	4.02	66.09
23	Fumaric acid	0	6.51	138.03
24	Glucose_1	0	3.23	76.85
25	Glucose_2	1	3.4	72.31
26	Glucose_3	2	3.45	78.69
27	Glucose_4	3	3.47	78.47
28	Glucose_5	4	3.71	63.45
29	Glucose_6	5	3.88	63.45
30	Glucose_7	6	4.64	98.64
31	Glutamic acid_1	0	2.08	29.73
32	Glutamic acid_2	1	2.34	36.2
33	Glutamic acid_3	2	3.75	57.36
34	Histidine_1	0	3.14	30.7
35	Histidine_2	1	3.23	30.7
36	Histidine_3	2	3.98	57.44

37	Histidine_4	3	7.07	119.57
38	Histidine_5	4	7.83	138.87
39	Lactic acid_1	0	1.32	22.81
40	Lactic acid_2	1	4.1	71.23
41	Leucine_1	0	0.94	23.59
42	Leucine_2	1	0.96	24.75
43	Leucine_3	2	1.7	26.87
44	Leucine_4	3	1.7	42.53
45	Leucine_5	4	3.72	56.11
46	Lysine_1	0	1.46	24.14
47	Lysine_2	1	1.72	29.14
48	Lysine_3	2	1.9	32.63
49	Lysine_4	3	3.01	41.76
50	Lysine_5	4	3.75	57.19
51	Succinic acid	0	2.39	36.92
52	Ribose 5 Phosphate_1	0	3.85	67.33
53	Ribose 5 Phosphate_1	1	3.91	67.33
54	Ribose 5 Phosphate_1	2	4.05	78.03
55	Ribose 5 Phosphate_1	3	4.07	84.63
56	Ribose 5 Phosphate_1	4	4.3	73.41
57	Ribose 5 Phosphate_1	5	5.22	103.82

Table S3. The list of metabolites in reference 2 model mixture.

No.	Metabolites/Conc. (μM)	Sample1	Sample2	Sample3	Sample4	Sample5	Sample6
1	Glutamic acid	2000	50	100	200	500	1000
2	AcetylCholine	100	200	500	1000	2000	50
3	Cysteine	200	500	1000	2000	50	100
4	Leucine	500	1000	2000	50	100	200
5	Alanine	1000	2000	50	100	200	500
6	Lysine	2000	50	100	200	500	1000
7	Ornithine	50	100	200	500	1000	2000
8	Arginine	100	200	500	1000	2000	50
9	Choline	200	500	1000	2000	50	100
10	Glutamine	200	500	1000	2000	50	100
11	n.a.	1000	2000	50	100	200	500
12	Succinic acid	2000	50	100	200	500	1000
13	Acetic acid	50	100	200	500	1000	2000
14	Lactic acid	100	200	500	1000	2000	50
15	Malic acid	200	500	1000	2000	50	100
16	Citric acid	500	1000	2000	50	100	200
17	Pyruvic acid	1000	2000	50	100	200	500
18	Acetyl-phosphate	2000	50	100	200	500	1000
19	Fumaric acid	50	100	200	500	1000	2000
20	2-HG	100	200	500	1000	2000	50
21	Glucose	500	1000	2000	50	100	200
22	AMP	500	1000	2000	50	100	200
23	NAD	1000	2000	50	100	200	500
24	Glucosamine	50	100	200	500	1000	2000
25	UDP	50	100	200	500	1000	2000
26	GTP	100	200	500	1000	2000	50
27	Ribose 5-phosphate	200	500	1000	2000	50	100
28	Histidine	500	1000	2000	50	100	200
29	Cytidine	1000	2000	50	100	200	500
30	Fructose	2000	50	100	200	500	1000

Table S4. Peak assignment for 29 metabolites in reference 2.

Peak #	Metabolites	Index	H (ppm)	C (ppm)
1	2-Hydroxyglutaric acid_1	0	1.82	33.7
2	2-Hydroxyglutaric acid_2	1	1.98	33.7
3	2-Hydroxyglutaric acid_3	2	2.25	36.03
4	2-Hydroxyglutaric acid_4	3	4.01	74.8
5	Acetic acid_1	0	1.9	25.99
6	Acetylphosphate_1	0	2.1	24.76
7	Acetylcholine_1	0	2.14	22.91
8	Acetylcholine_2	1	3.21	56.46
9	Acetylcholine_3	2	3.73	67.2
10	Acetylcholine_4	3	4.55	61.05
11	Alanine_1	0	1.47	18.87
12	Alanine_2	1	3.77	53.22
13	AMP_1	0	4.04	66.24
14	AMP_2	1	4.38	87.42
15	AMP_3	2	4.53	73.45
16	AMP_4	3	4.79	77.29
17	AMP_5	4	6.09	89.64
18	AMP_6	5	8.11	155.34
19	AMP_7	6	8.56	142.77
20	Arginine_1	0	1.65	26.58
21	Arginine_2	1	1.71	26.58
22	Arginine_3	2	1.91	30.28
23	Arginine_4	3	3.24	43.2
24	Arginine_5	4	3.76	57
25	Choline_1	0	3.19	56.58
26	Choline_2	1	3.51	70.12
27	Choline_3	2	4.05	58.33
28	Citrate_1	0	2.52	48.45
29	Citrate_2	1	2.64	48.45
30	Cysteine_1	0	3.02	27.65
31	Cysteine_2	1	3.07	27.65
32	Cysteine_3	2	3.95	58.68
33	Cytidine_1	0	3.81	63.42
34	Cytidine_2	1	3.92	63.42
35	Cytidine_3	2	4.12	86.48
36	Cytidine_4	3	4.2	71.98

37	Cytidine_5	4	4.3	76.69
38	Cytidine_6	5	5.89	92.98
39	Cytidine_7	6	6.03	98.96
40	Cytidine_8	7	7.84	144.33
41	Fructose_1	0	3.56	66.57
42	Fructose_2	1	3.7	66.09
43	Fructose_3	2	3.71	66.57
44	Fructose_4	3	3.79	70.23
45	Fructose_5	4	3.89	72.39
46	Fructose_6	5	3.99	71.93
47	Fructose_7	6	4.02	66.09
48	Fumaric acid_1	0	6.51	138.03
49	Glucosamine_1	0	3.2	57.14
50	Glucosamine_2	1	3.46	72.33
51	Glucosamine_3	2	3.83	63.08
52	Glucosamine_4	3	3.84	72.94
53	Glucosamine_5	4	3.87	74.35
54	Glucosamine_6	5	5.41	92.42
55	Glucose_1	0	3.23	76.85
56	Glucose_2	1	3.4	72.31
57	Glucose_3	2	3.45	78.69
58	Glucose_4	3	3.47	78.47
59	Glucose_5	4	3.71	63.45
60	Glucose_6	5	3.88	63.45
61	Glucose_7	6	4.64	98.64
62	Glutamic acid_1	0	2.08	29.73
63	Glutamic acid_2	1	2.34	36.2
64	Glutamic acid_3	2	57.36	3.77
65	Glutamine_1	0	2.13	28.95
66	Glutamine_2	1	2.44	33.52
67	Glutamine_3	2	3.76	56.83
68	GTP_1	0	4.2	67.72
69	GTP_2	1	4.25	67.72
70	GTP_3	2	4.35	86.65
71	GTP_4	3	4.59	73.03
72	GTP_5	4	4.79	76.35
73	GTP_6	5	5.92	89.3
74	GTP_7	6	8.13	140.43
75	Histidine_1	0	3.14	30.7

76	Histidine_2	1	3.23	30.7
77	Histidine_3	2	3.98	57.44
78	Histidine_4	3	7.07	119.57
79	Histidine_5	4	7.83	138.87
80	Lactic acid_1	0	1.32	22.81
81	Lactic acid_2	1	4.1	71.23
82	Leucine_1	0	0.94	23.59
83	Leucine_2	1	0.96	24.75
84	Leucine_3	2	1.7	26.87
85	Leucine_4	3	1.7	42.53
86	Leucine_5	4	3.72	56.11
87	Lysine_1	0	1.46	24.14
88	Lysine_2	1	1.72	29.14
89	Lysine_3	2	1.9	32.63
90	Lysine_4	3	3.01	41.76
91	Lysine_5	4	3.75	57.19
92	Malic acid_1	0	2.36	45.32
93	Malic acid_2	1	2.66	45.32
94	Malic acid_3	2	4.29	73.06
95	NAD_1	0	4.26	67.65
96	NAD_2	1	4.26	68.18
97	NAD_3	2	4.38	67.65
98	NAD_4	3	4.4	86.51
99	NAD_5	4	4.45	73.28
100	NAD_6	5	4.5	80.33
101	NAD_7	6	4.53	73.11
102	NAD_8	7	4.56	89.67
103	NAD_9	8	4.77	76.7
104	NAD_10	9	5.99	89.39
105	NAD_11	10	6.1	102.74
106	NAD_12	11	8.02	155.33
107	NAD_13	12	8.2	131.33
108	NAD_14	13	8.38	142.3
109	NAD_15	14	8.82	148.43
110	NAD_16	15	9.15	145.05
111	NAD_17	16	9.34	142.65
112	Ornithine_1	0	1.8	25.47
113	Ornithine_2	1	1.94	30.18
114	Ornithine_3	2	3.04	41.56

115	Ornithine_4	3	3.77	56.8
116	Pyruvic acid_1	0	2.36	29.2
117	Ribose 5-phosphate_1	0	3.85	67.33
118	Ribose 5-phosphate_2	1	3.91	67.33
119	Ribose 5-phosphate_3	2	4.05	78.03
120	Ribose 5-phosphate_4	3	4.07	84.63
121	Ribose 5-phosphate_5	4	4.3	73.41
122	Ribose 5-phosphate_6	5	5.22	103.82
123	Succinic acid_1	0	2.39	36.92
124	UDP_1	0	4.22	66.8
125	UDP_2	1	4.26	85.86
126	UDP_3	2	4.38	76.59
127	UDP_4	3	4.43	71.82
128	UDP_5	4	5.95	91.44
129	UDP_6	5	5.97	105.23
130	UDP_7	6	7.99	144.52

Table S5. The peaks that were used to calculate R^2, LOD and LOQ.

1	2	3	4	5	6	7
UDP_1	UDP_2	UDP_3	UDP_4	UDP_5	UDP_6	UDP_7
Cytidine_2	Cytidine_3	Cytidine_5	Cytidine_6	Cytidine_7	Cytidine_8	
Fructose_1	Fructose_2	Fructose_4	Fructose_5	Fructose_6	Fructose_7	
Ribose 5P _1	Ribose 5P _2	Ribose 5P _3	Ribose 5P _4	Ribose 5P _5	Ribose 5P _6	
NAD_3	NAD_5	NAD_6	NAD_7	NAD_8	NAD_11	NAD_13
NAD_14	NAD_15	NAD_16	NAD_17			
AMP_1	AMP_2	AMP_4	AMP_5	AMP_7		
Glucose_1	Glucose_3	Glucose_4	Glucose_5	Glucose_7		
Histidine_1	Histidine_2	Histidine_3	Histidine_4	Histidine_5		
2-HG_1	2-HG _2	2-HG _3	2-HG _4			
GTP_1	GTP_4	GTP_6	GTP_7			
Leucine_1	Leucine_2	Leucine_4	Leucine_5			
Acetylcholine_1	Acetylcholine_3	Acetylcholine_4				
Cysteine_1	Cysteine_2	Cysteine_3				
Glucosamine_4	Glucosamine_5	Glucosamine_6				
Lysine_1	Lysine_2	Lysine_3				
Malic acid_1	Malic acid_2	Malic acid_3				
Alanine_1	Alanine_2					
Arginine_1	Arginine_4					
Choline_2	Choline_3					

Glutamic acid_1	Glutamic acid_2					
Glutamine_1	Glutamine_2					
Lactic acid_1	Lactic acid_2					
Ornithine_1	Ornithine_3					
Citrate_2						
Acetic acid_1						
Acetylphosphate_1						
Fumaric acid_1						
Pyruvic acid_1						
Succinic acid_1						

Table S6. The peaks that were missing in 50% and 25% NUS compared to US spectrum.

Peak #	H	C	100%	50%	25%
1	7.178	133.267	1.50E+06	2.35E+06	3.28E+06
2	7.328	131.882	1.66E+06	1.50E+06	N
3	7.43	131.359	8.13E+05	N	N
4	6.872	118.642	1.66E+06	3.34E+06	1.75E+06
5	4.666	98.413	3.32E+07	7.01E+07	6.44E+07
6	5.251	94.613	2.63E+07	5.70E+07	5.21E+07
7	4.297	84.474	8.91E+05	N	N
8	4.839	84.344	8.64E+05	N	N
9	3.495	78.418	4.40E+07	8.42E+07	7.74E+07
10	3.423	78.424	1.17E+07	2.28E+07	2.02E+07
11	4.175	78.032	9.73E+05	N	N
12	4.668	76.625	1.03E+06	2.33E+06	2.09E+06
13	3.264	76.621	3.23E+07	6.37E+07	6.08E+07
14	3.659	75.424	1.69E+06	2.86E+07	N
15	3.725	75.338	1.47E+07	2.86E+07	2.67E+07
16	3.846	74.015	1.93E+07	3.88E+07	3.54E+07
17	3.555	74.004	1.99E+07	4.13E+07	3.78E+07
18	3.852	72.174	1.80E+06	2.07E+06	1.64E+06
19	3.728	72.18	1.66E+06	2.56E+06	2.51E+06
20	3.424	72.11	3.53E+07	7.15E+07	6.49E+07
21	3.498	72.002	4.18E+06	8.27E+06	6.00E+06
22	3.606	71.644	1.46E+06	1.59E+06	1.67E+06
23	4.004	71.26	1.22E+06	1.88E+06	N
24	3.954	71.224	9.52E+05	1.88E+06	N
25	3.852	71.116	1.05E+06	N	N
26	3.591	71.127	1.42E+06	1.59E+06	1.67E+06
27	4.125	71.071	1.47E+07	2.93E+07	2.63E+07
28	1.335	70.988	8.21E+05	2.14E+06	N
29	3.704	70.917	1.15E+06	1.81E+06	N
30	4.157	68.155	1.10E+06	N	N
31	3.806	66.175	1.20E+06	N	N
32	3.665	65.168	3.19E+06	5.00E+06	3.84E+06
33	3.577	65.076	8.85E+05	N	N
34	3.139	63.527	1.95E+06	3.71E+06	2.29E+06
35	3.489	63.444	1.15E+06	1.73E+06	1.86E+06
36	3.427	63.441	1.59E+06	2.47E+06	1.86E+06
37	3.743	63.31	2.57E+07	5.10E+07	4.60E+07

38	3.908	63.273	2.69E+07	5.45E+07	4.86E+07
39	3.849	63.16	1.68E+07	3.39E+07	3.13E+07
40	3.56	63.167	2.53E+06	4.06E+06	3.49E+06
41	3.946	59.009	1.58E+06	2.01E+06	2.10E+06
42	4.071	58.947	9.76E+05	N	1.54E+06
43	3.851	58.96	9.94E+05	N	N
44	2.575	57.681	1.79E+06	2.67E+06	2.00E+06
45	3.676	57.354	2.04E+06	3.99E+06	3.58E+06
46	3.623	57.194	4.90E+06	7.47E+06	8.29E+06
47	3.235	56.631	5.32E+06	8.75E+06	6.51E+06
48	3.944	56.395	9.29E+05	N	N
49	3.702	55.986	9.45E+05	2.17E+06	3.58E+06
50	3.284	55.937	3.49E+06	5.81E+06	4.99E+06
51	3.823	54.558	1.05E+06	N	N
52	3.764	53.251	2.03E+06	3.15E+06	1.88E+06
53	3.369	51.487	5.04E+07	1.05E+08	1.01E+08
54	2.322	49.177	1.10E+06	N	N
55	3.535	44.427	3.06E+06	5.24E+06	4.76E+06
56	3.249	43.205	1.97E+06	3.10E+06	2.63E+06
57	1.672	42.776	7.92E+05	N	N
58	1.718	42.638	1.07E+06	N	N
59	3.029	41.719	4.67E+06	8.32E+06	6.84E+06
60	2.904	41.632	1.09E+06	N	2.62E+06
61	2.335	36.17	2.02E+06	2.02E+06	2.02E+06
62	2.328	34.364	1.90E+06	2.36E+06	1.69E+06
63	2.421	33.707	6.32E+06	1.09E+07	1.13E+07
64	1.865	33.222	2.04E+06	2.74E+06	2.27E+06
65	2.052	31.871	1.21E+06	1.73E+06	N
66	1.852	31.402	1.78E+06	2.86E+06	N
67	2.057	30.273	4.35E+06	6.60E+06	5.70E+06
68	1.727	29.099	2.40E+06	4.05E+06	3.45E+06
69	1.48	27.119	9.66E+05	N	N
70	1.651	26.922	9.53E+05	N	N
71	1.714	26.7	1.25E+06	2.00E+06	2.05E+06
72	2.027	26.388	1.02E+06	N	N
73	1.931	25.907	1.50E+06	2.25E+06	1.74E+06
74	2.056	24.773	5.71E+06	1.04E+07	1.05E+07
75	0.969	24.599	2.96E+06	5.52E+06	5.12E+06
76	1.504	24.278	1.21E+06	1.69E+06	1.57E+06

77	1.452	24.278	1.21E+06	1.69E+06	1.44E+06
78	0.96	23.756	2.60E+06	4.06E+06	3.14E+06
79	0.903	23.693	9.61E+05	N	N
80	1.057	22.619	9.72E+05	N	1.57E+06
81	1.225	21.536	1.52E+06	4.41E+06	N
82	0.939	21.319	7.22E+05	1.76E+06	N
83	0.977	21.041	9.61E+05	1.76E+06	N
84	1.041	20.693	4.49E+06	7.91E+06	6.66E+06
85	0.988	19.232	4.44E+06	8.28E+06	7.89E+06
86	1.472	19.085	8.42E+06	1.58E+07	1.44E+07
87	1.014	17.251	1.39E+06	1.58E+06	1.47E+06
88	0.796	16.616	1.16E+06	1.61E+06	N
89	0.943	13.801	1.03E+06	N	N
90	1.359	4.288	1.53E+06	N	N
91	0.057	3.279	7.08E+06	7.08E+06	7.08E+06
92	1.205	24.253	2.77E+06	6.51E+06	4.84E+06
93	1.351	23.008	4.03E+07	1.29E+08	1.22E+08
94	1.295	21.607	2.64E+06	4.41E+06	3.11E+06
95	1.261	22.619	1.08E+06	2.01E+06	2.83E+06
96	2.231	32.344	1.03E+06	N	1.63E+06
97	3.367	83.332	1.11E+06	2.52E+06	3.85E+06
98	3.919	74.884	5.79E+05	3.26E+06	3.54E+06
99	4.305	61.913	8.02E+05	N	N
100	3.894	65.505	1.71E+06	3.25E+06	3.79E+06
101	3.484	80.605	3.17E+05	N	1.73E+06
102	3.79	74.818	1.02E+06	N	2.29E+06
